# Autonomous scanning probe microscopy with hypothesis learning: Exploring the physics of domain switching in ferroelectric materials

**DOI:** 10.1016/j.patter.2023.100704

**Published:** 2023-03-10

**Authors:** Yongtao Liu, Anna N. Morozovska, Eugene A. Eliseev, Kyle P. Kelley, Rama Vasudevan, Maxim Ziatdinov, Sergei V. Kalinin

**Affiliations:** 1 Center for Nanophase Materials Sciences, Oak Ridge National Laboratory, Oak Ridge, TN 37922, USA; 2 Institute of Physics, National Academy of Sciences of Ukraine, 46, pr. Nauky, 03028 Kyiv, Ukraine; 3 Institute for Problems of Materials Science, National Academy of Sciences of Ukraine, Krjijanovskogo 3, 03142 Kyiv, Ukraine; 4 Computational Sciences and Engineering Division, Oak Ridge National Laboratory, Oak Ridge, TN 37831, USA

**Keywords:** hypothesis learning, automated experiment, scanning probe microscopy, ferroelectrics

## Abstract

Using hypothesis-learning-driven automated scanning probe microscopy (SPM), we explore the bias-induced transformations that underpin the functionality of broad classes of devices and materials from batteries and memristors to ferroelectrics and antiferroelectrics. Optimization and design of these materials require probing the mechanisms of these transformations on the nanometer scale as a function of a broad range of control parameters, leading to experimentally intractable scenarios. Meanwhile, often these behaviors are understood within potentially competing theoretical hypotheses. Here, we develop a hypothesis list covering possible limiting scenarios for domain growth in ferroelectric materials, including thermodynamic, domain-wall pinning, and screening limited. The hypothesis-driven SPM autonomously identifies the mechanisms of bias-induced domain switching, and the results indicate that domain growth is ruled by kinetic control. We note that the hypothesis learning can be broadly used in other automated experiment settings.

## Highlights

• Hypothesis learning combines machine learning with physical hypotheses
• Hypothesis learning enables physical discovery in the experiment
• Hypothesis learning is implemented in operating scanning probe microscopy
• Hypothesis learning identifies the ferroelectric domain growth mechanism autonomously

## The bigger picture

The need for automated experimentation has been recently recognized across multiple areas of instrument-based sciences, including X-ray scattering, electron, and scanning probe microscopy, necessitating the development of algorithms for navigating multidimensional compositional or processing spaces. Autonomous discovery of physical laws via experiment has emerged as a rapidly emerging topic for machine learning in physical sciences. Here, we report the development of a hypothesis-learning-based automated experiment, which autonomously identifies the mechanism behind materials’ response during operating experiments. This approach can be applied to a broad range of physical and chemical experiments with relatively low-dimensional control parameter space and for which the possible competing models of the system behavior that ideally cover the full range of physical eventualities are known.

## Graphical abstract

**Figure:**
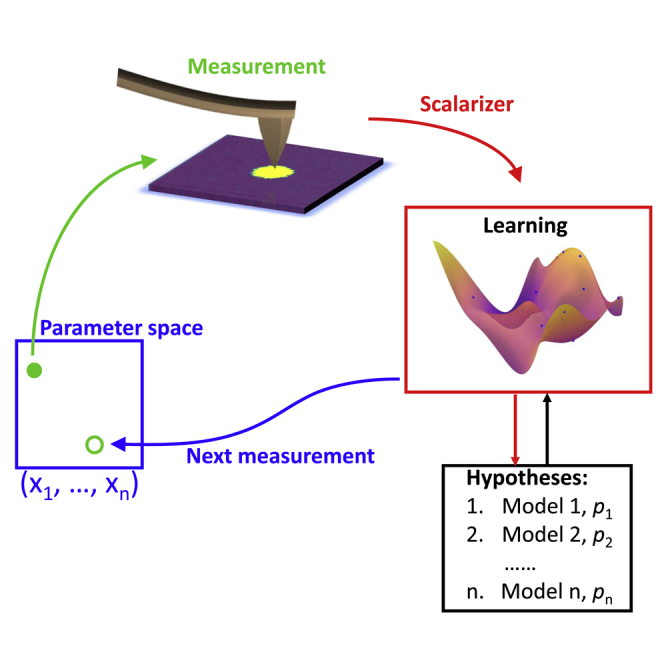

## Teaser

Hypothesis-learning-driven automated scanning probe microscopy allows physical discovery during experiments, where the hypothesis learning algorithm implemented on an operating microscope identifies the physical mechanism behind the material’s response.

## Introduction

Machine learning (ML) methods are rapidly becoming an inseparable part of physical sciences, with the applications ranging from astronomy^1^^,^^2^ and high energy physics^3^ to materials science^4^^,^^5^^,^^6^ and microscopy.^7^ While early demonstrations of ML have been shown in a variety of domain disciplines including computational sciences and first-principles theory,^8^^,^^9^ microscopy,^10^ materials growth,^11^ theory-experiment matching,^12^ and many others, these efforts remained largely isolated. In theory and computation, the watershed moment was the publication of the seminal 2006 paper by Ceder^13^ that launched the materials genome project and subsequently gave rise to multiple ML efforts. The second key point was the demonstration of deep learning by Krizhevsky^14^ that ushered in the deep-learning era of today and sparked enthusiasm and investment toward ML applications across multiple disciplines. Currently, the most effort in the ML field is concentrated on classical big data approaches. Applications such as GPT and DALL-E have captured the attention of professionals and the general community.^15^^,^^16^

However, despite their power for image analysis and natural language applications, these big data methods are remarkably limited in domain applications, where the volumes of data sufficient to sample the distribution are rare. In the correlative models, complications arise due to the out-of-distribution shift effects, which are explored both in model examples and in applications in medical imaging and automated driving. Secondly, compared with classical ML problems, physical sciences offer a challenge of active learning with very limited experimental budget. However, it is also well understood that in physical sciences, the nature of scientific domain offers a rich set of prior knowledge in forms ranging from known physical laws and constraints and expected parameter values to difficult-to-define physical intuition.

Particularly of interest are the applications of active learning where the ML agent interacts with a physical object via a suitable measurement tool, i.e., an automated experiment. The need for automated experimentation has been recently recognized across multiple areas of instrument-based sciences, including X-ray scattering, electron, and scanning probe microscopy.^17^^,^^18^^,^^19^ Similarly, rapid growth in automated synthesis platforms, including computer-controlled synthesis,^20^ fully automated labs,^21^ microfluidic systems,^22^ and combined human-high-throughput experimentation workflows,^23^ necessitates the development of algorithms for navigating multidimensional compositional or processing spaces. Notably, the (initial) requirements for automated experimentation in microscopy and synthesis are close and allow for the use of classes of algorithms based on Bayesian optimization (BO).^24^^,^^25^^,^^26^

The important limitation of the classical BO strategies with the Gaussian process is the use of the non-parametric kernel-based models. In this case, the internal correlations across the data space are used to select the locations for a new experiment. However, these models do not contain any specific physical assumptions or relationships. Hence, in many cases, the efficiency of BO-based methods is only within an order of magnitude from classical grid search-based strategies. At the same time, often, the behaviors of these systems are understood within potentially competing theoretical models or hypotheses.

Recently, we have introduced the approach for physics-informed BO in automated experiments, referred to as hypothesis learning.^27^ In this approach, a list of possible models (hypotheses) of the system behavior is established prior to the automated experiment. The hypotheses in this case are the analytical expressions (or other fast computational schemes), with partial knowledge of the associated parameters in the form of Bayesian priors formed based on the analysis of physics of observed phenomena. During the experiment, the algorithm aims to narrow down the range of possible hypotheses following a certain optimization policy, i.e., it tries to establish the best model of the system’s behavior within the smallest number of steps. The thus identified model will represent the mechanism of the observed physical phenomena. Ideally, the list of hypotheses will enumerate possible scenarios for materials behavior; however, if the correct model is not a part of the list, the algorithm reverts to the structured model closest to the ground-truth behavior or adopts a structureless Gaussian prior. In this manner, the algorithm discovers the physical laws operating in the studied system.

Here, we illustrate the hypothesis-learning-based automated experiment for the explorations of the domain-switching mechanisms in classical ferroelectric materials. While shown for a model system, this approach is more general and can be broadly used for exploration of other scanning probe microscopy (SPM)-based electrochemical reactions and for automated experimentation in scattering, microscopy, and materials synthesis.

## Results and discussion

### Principles of hypothesis learning

The bedrock element of physical sciences is the validated set of quantitative symbolic relationships between the physical parameters, numerical constants, and observables. Examples range from the fundamental laws of the Newtonian mechanics to expressions defining current-voltage relationships in semiconductors. In certain cases, these relationships are derived from fundamental laws and symmetries. In others, they represent a useful empirical generalization, valid under specific conditions. Using these relationships underpins all areas of physics research, and deriving these relationships is often equated with the understanding of the relevant physical mechanisms.

The research process often involves iterative cycles between the acquisition of the experimental data and their interpretation in terms of specific models. In some cases, the underlying symbolic relationships are discovered via exploratory data analysis, with the subsequent interpretation based on the functional form of derived relationships. In other cases, a number of competing models can be derived based on prior knowledge and fundamental physical laws, and the model best matching the experiment is selected to represent the relevant physics. In all cases, models include not only the symbolic form, per se, but also the expected values of the internal parameters that are known with different degree of certainty, naturally cast in the Bayesian inference (BI) framework.

**Figure 1:**
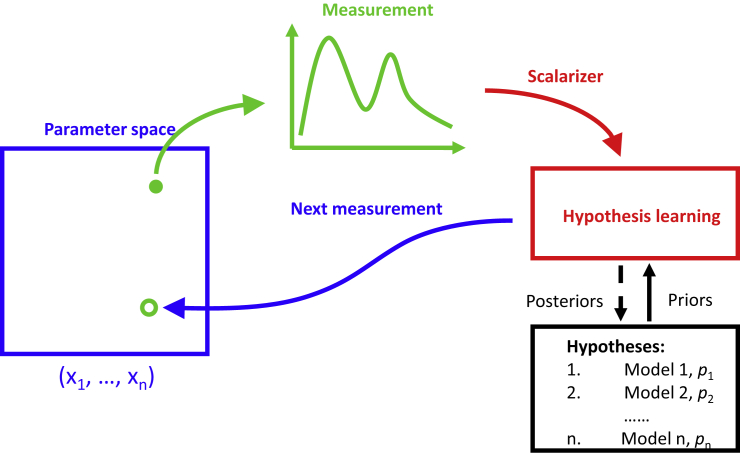
Schematics of the hypothesis learning in automated experiment The measurement is performed in a selected location(s) in the parameter space. These can be control parameters of experiment, concentrations in the phase diagram, or image plane. The measurement result is converted to a scalar measure of interest. The hypothesis-learning agent uses the measurement to establish the posterior probabilities of a sampled model and select next location(s) in parameter space for measurements. Note that models corresponding to different physical scenarios (hypotheses) return the scalar values derived via the same scalarizer as the experiment.

Hypothesis learning is developed as an approach to implement this iterative cycle as a part of the automated experiment (Figure 1).^27^ Here, the automated experiment generally refers to sequential (or batch) measurements of a target functionality over pre-defined parameter space. The scalarizer function reduces the (potentially vector-valued) functionality to a single scalar, defined to represent a measure of the experimentalist’s interest in a specific physical property or response. Several possible hypotheses describing the system’s behavior are available to complement an automated experiment. The hypothesis generally refers to a model predicting the functionality of interest (or its scalarized form) over the parameter space. Ideally, the list of models reflects the full list of possible mechanisms active within the material and fully covers the possible physical scenarios. The key requirement of the model is the ease of calculation, as is necessary to perform Bayesian evaluations based on Markov chain Monte Carlo techniques. Here, for convenience, we use the hypotheses in the symbolic equation form; however, this requirement can be relaxed to numerical models. The symbolic expression and associated prior distributions of parameters define a single hypothesis. A list of possible hypotheses with associated prior probabilities (which may or may not include the ground truth one) is a second component of hypothesis learning.

During the hypothesis learning, the agent performs the experiment, returning the scalarized value of the functionality of interest in a selected point of the parameter space. The measured values are used to perform the BI on the list of probabilistic models (hypotheses) wrapped into the structured Gaussian processes,^28^ generating posterior probabilities of the models’ parameters. The latter is used to obtain posterior predictive uncertainties over the unmeasured points of the parameters space. The model that produced the lowest predictive uncertainty is assigned a positive reward value and is used to sample the next measurement point according to a pre-defined acquisition function. Because running BI for every model on the list at each step is computationally expensive, we do it only for several steps (“warm-up phase”) and then switch to the epsilon-greedy policy for sampling a single model (hypothesis) at each step. If the sampled model reduces/increases the predictive uncertainty compared with the previous step, it receives a positive/negative reward.

### Mechanisms for ferroelectric domain growth

The phenomenological mechanisms for ferroelectric domain growth in piezoresponse force microscopy (PFM) have been extensively explored for over two decades.^29^^,^^30^^,^^31^ The early studies have established the phenomenological relationship between the size of the formed domain and the parameters of the bias pulse applied to the tip.^32^^,^^33^^,^^34^^,^^35^ The initial theoretical analyses of the domain switching were based on either purely thermodynamic considerations in the rigid ferroelectric,^36^^,^^37^^,^^38^ Ginzburg-Landau approximations,^39^^,^^40^ or analysis of the domain wall motion in the electrostatic field of the probe.^35^^,^^41^^,^^42^ At the same time, these analyses have demonstrated that thermodynamics of polarization switching strongly depends on the effectiveness of the screening process on the top surface. Correspondingly, the kinetics of the process can be limited by the screening process rather than intrinsic material behavior. From the experimental perspective, ample evidence exists toward the role of screening charge dynamics in switching through observations of charge injection,^43^^,^^44^ back switching and formation of bubble domains,^45^^,^^46^^,^^47^^,^^48^^,^^49^ chaotic switching dynamics^50^ and formation of complex domains,^51^ vortices, and skyrmions.^52^^,^^53^^,^^54^

Hence, despite its apparent simplicity, domain switching in ferroelectrics is a complex process that is affected by the intrinsic thermodynamics of the domain formation, domain wall pinning in the spatially non-uniform probe field, and screening charge generation and dynamics. Any of these can serve as a process limiting stage, forming an ideal setting for hypothesis-learning applications. Importantly, at the mesoscopic level, these mechanisms provide the full range of possible physical eventualities, and thus the hypothesis list is assumed to be complete.

**Figure 2:**
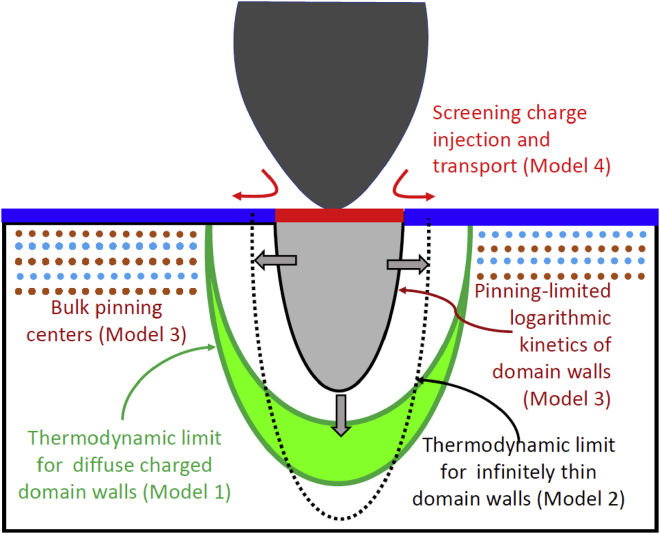
Schematics of the domain nucleation models Model I shows thermodynamic limit for diffuse domain walls, which are thicker when becomes charged. Model II shows thermodynamic limit for infinitely thin domain walls. Model III is limited by domain wall pining in the bulk, giving rise to the logarithmic kinetics. Model IV is limited by the injection and transport of surface charged species that are necessary for the polarization screening.

Here, we enumerate the hypotheses for the domain growth based on (1) thermodynamic control in the presence or absence of surface screening charges, (2) kinetic control of domain wall motion via pinning, or (3) kinetic control via screening charge dynamics. This list of possible limiting factors is exhaustive for the domain formation in PFM, and hence the selected hypotheses are expected to cover the full list of experimental eventualities. The analysis of the laws of domain growth in these cases was carried out by a number of groups over the last two decades,^34^^,^^55^^,^^56^^,^^57^^,^^58^^,^^59^ and below we give only corresponding approximate expressions. The detailed voltage dependences of the equilibrium domain sizes (1–3) are described in details in Appendix A. Figure 2 illustrates schematically the domain nucleation models.

Here, model I corresponds to the partial internal screening of the depolarization field by space charge carriers and domain wall thickening. For this case, the equilibrium domain radius depends on the tip voltage as

(Equation 1) $$r\left(V\right)\approx r_{cr}+d\sqrt{{\left(\frac{V}{V_{C}}\right)}^{2/3}-1}\text{,}$$

meaning a jump at $V_{c}$ corresponding to the first-order phase transition and further growth as roughly as ${\left(V/V_{c}\right)}^{1/3}$. For complete screening, the jump disappears, and the equilibrium domain radius depends on the tip voltage as $r\left(V\right)\approx d\sqrt{{\left(V/V_{c}\right)}^{2/3}-1}$, i.e., as the second-order phase transition scenario. Note that in the Bayesian setting, these models are similar and can be defined by tuning the prior distributions on parameter *r*_*cr*_.

Model II corresponds to a Landauer-Molotskii (LM) approach of infinitely thin domain walls, very prolate domains, and their breakdown (see Figure 2).^60^^,^^61^ For this case, the equilibrium domain radius depends on the tip voltage as

(Equation 2) $$r\left(V\right)=r_{cr}+r_{0}\sqrt[{3}]{{\left(\frac{V}{V_{c}}\right)}^{2}-1}\text{.}$$

Again, a jump $V_{c}$ corresponds to the first-order phase transition and further growth as ${\left(\frac{V}{V_{c}}\right)}^{2/3}$.

Model III: alternative to the thermodynamic control of the domain size is the kinetic control, in which case the domain size is determined by the kinetics of the domain wall motion. From general theory of the disordered media, the domain wall velocity in the uniform field follows the classical dependence including pinning, creep, and then depinning and linear motion.^33^^,^^62^^,^^63^^,^^64^ The available kinetic models^42^^,^^65^ for the domain wall velocity v(r) in a ferroelectric domain with pinning relate an acting electric field E, a threshold field $E_{th}$, and as $v\left(r\right)\approx v_{0}\;\exp\left[-{\left(E_{th}/E\right)}^{\mu }\right]$, where μ is a positive exponential factor, which is typically close to unity. Using the simplest form for a normal component of the tip field, $E_{z}\left(r,0\right)=\frac{Vd^{2}}{\gamma {\left(r^{2}+d^{2}\right)}^{3/2}}$, where γ is a dielectric anisotropy factor, *d* is the effective tip size, r is a surface distance from the tip axis, and V is the bias applied between the tip and the bottom electrode, the approximate solution for the time dependence of the domain radius can be derived as (Appendix A)

(Equation 3) $$r\left(t\right)\approx {\left(\frac{V}{\beta }\right)}^{1/3}\ln\left[1+{\left(\frac{\beta }{V}\right)}^{1/3}v_{0}t\right]\text{,}$$

where the parameter $\beta ={\gamma E}_{th}/d^{2}$. Expression (4) describes a slow logarithmic creep of the domain wall such that $r\left(V\right)∼V^{1/3}$ at high voltages. For ${\left(\beta /V\right)}^{1/3}v_{0}t\gg 1$, we obtain $r\left(t\right)∼{\left(V/\beta \right)}^{1/3}\ln\left[v_{0}t\right]$. However, the lateral growth stops at equilibrium domain sizes after the pulse ending, which can be calculated from thermodynamic description.

Finally, as model IV, we consider the case where the domain growth is limited by the transport of the screening charges across the sample surface. Here, we note that, in general, polarization switching requires almost complete compensation of the polarization charges by screening charges. If screening charges are abundant, the domain is determined by switching thermodynamics (model I and II) or wall pinning (model III). If the screening charges are slow and sparse, then the domain growth is limited by the charge injection. The experimental evidence toward this behavior was obtained by Yudin et al.^35^ and also indirectly via observations of phenomena such as chaotic domain switching.^50^^,^^51^

The simple consideration of the mass and charge balance suggest that in the PFM experiment, the screening charges can be generated only at the tip-surface junction. In this case, assuming general power law voltage dependence of the generation rate and diffusional or drift transport of charge species, the kinetics of the domain wall growth can be described as

(Equation 4) $$r\left(\tau \right)\approx V^{\alpha }\tau^{\beta }\text{,}$$

where, in the depletion approximation, α is close to 1 and β is 1/2.

### Experimental realization of hypothesis learning

**Figure 3:**
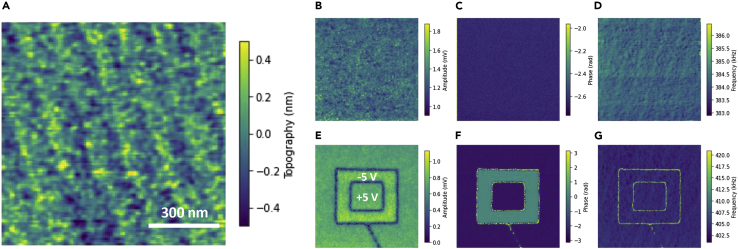
BEPFM of BTO sample (A) Topography with periodical terrace structure. (B–D) BEPFM amplitude, phase, and frequency images of the pristine state. (E–G) BEPFM amplitude, phase, and frequency images of pre-poled areas by 5 V DC bias.

As a model ferroelectric system, we use a fully relaxed 80 nm thick BaTiO_3_ (BTO) thin film (see experimental procedures). A representative PFM image and domain writing are shown in Figure 3. Figure 3A is the surface topography showing uniform geometry with periodical terrace structures. Figures 3B and 3C are out-of-plane band-excitation PFM (BEPFM) amplitude and phase images of the original sample, respectively, and Figure 3D shows the corresponding resonance frequency related to local elastic property. The BTO film shows a down-polarized pristine state (Figure 3C). The polarization can be switched by applying a direct current (DC) bias via atomic force microscopy (AFM) tip. Shown in Figures 3E–3G are out-of-plane BEPFM amplitude, phase, and frequency images showing pre-poled areas by applying 5 V DC bias via AFM tip.

**Figure 4:**
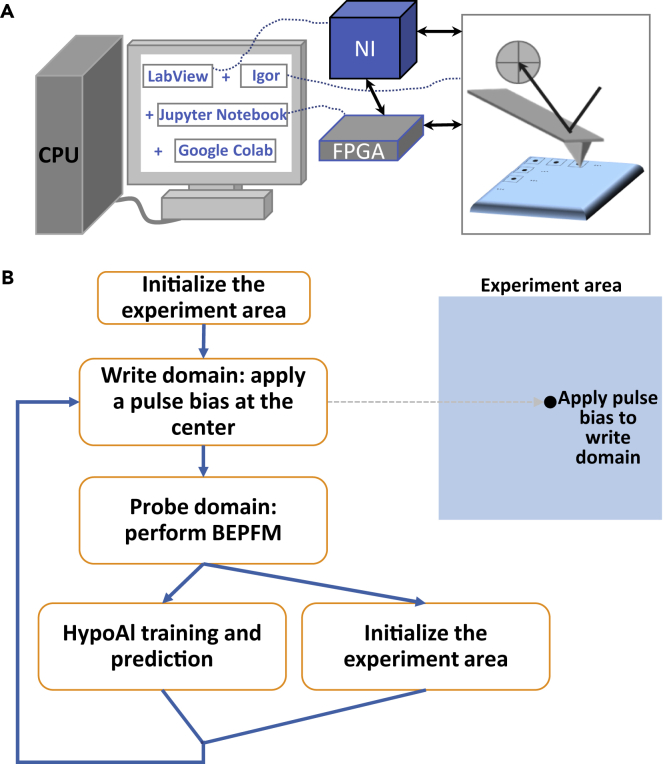
Hypothesis-learning automated BEPFM system and workflow (A) A schematic showing the integrated system for hypothesis-learning automated BEPFM. (B) Hypothesis-learning-based automated BEPFM workflow.

To realize hypothesis-learning-based automated experiment, we developed and deployed a workflow shown in Figure 4A integrating multiple software (including LabView, Jupyter Notebook, Google Colaboratory, and Igor) and hardware (including a National Instruments DAQ card, field-programmable gate arrays [FPGAs], and an Asylum Research Cypher microscope). To perform a BEPFM measurement, the measurement location (equivalent to tip location) is controlled by FPGAs, and BEPFM data are acquired by the National Instruments DAQ card. To apply a pulse bias for writing domain, FPGA moves the tip to the target location (center of the experiment area) and applies the pulse bias to tip.

The workflow of hypothesis-learning automated BEPFM is shown in Figure 4B. The experiment starts with initializing the measurement area by applying a DC bias to uniformly pole this area toward the same direction. Then, domain writing and imaging are performed in this area. The acquired BEPFM data are analyzed by a threshold filter to detect the written domain, and the domain size is determined by the minimum closed circle in which the written domain lies either inside the circle or on its boundaries. Next, this domain size and writing parameters, as well as all previous domain sizes and writing parameters, are fed to the hypothesis-learning model to predict the next writing parameters. Simultaneously, when the hypothesis-learning algorithm is performing training and prediction, the workflow also controls the microscope to back switch the measurement area (erase the domain) in order to be ready for next writing iteration. Then, the predicted writing parameters will be fed to the measurement workflow, and the writing/imaging process will be performed again. For this workflow, we also added a function to check the BEPFM data quality such that a poor dataset will be discarded, and the same measurement will be repeated. This checking process ensures that all BEPFM data and, consequently, domain sizes are comparable.

For the hypotheses list, we have chosen four models following our discussion in mechanisms for ferroelectric domain growth. Equations corresponding to these models are shown in Table 1.

**Table 1:** Model equations and priors used in the hypotheses-driven automated experiment

Model	Model equation	Model priors
I	$r\left(V\right)=r_{cr}+r_{0}\sqrt{{\left(\frac{V}{V_{c}}\right)}^{2/3}-1}$	$r_{cr}∼Normal\left(0,1\right)$ $r_{0}∼LogNormal\left(0,1\right)$ $V_{c}∼LogNormal\left(0,1\right)$
II	$r\left(V\right)=r_{cr}+r_{0}\sqrt[{3}]{{\left(\frac{V}{V_{c}}\right)}^{2}-1}$	$r_{cr}∼Normal\left(0,1\right)$ $r_{0}∼LogNormal\left(0,1\right)$ $V_{c}∼LogNormal\left(0,1\right)$
III	$r\left(V,t\right)=V^{\alpha }\;\log\;t$	α∼Uniform(0.33,1.2)
IV	$r\left(V,t\right)=V^{\alpha }t^{\beta }$	α∼Uniform(0.8,1.2) β∼Uniform(0.33,1.2)

In all models, *r* is the domain radius (“size”), *V* is voltage applied through tip; t is the time of applied bias; and $V_{c}$, $r_{cr}$, $r_{0}$, α, and β are the model parameters, which will be inferred during experiments. More details about these models are available in Appendix S1.

**Figure 5:**
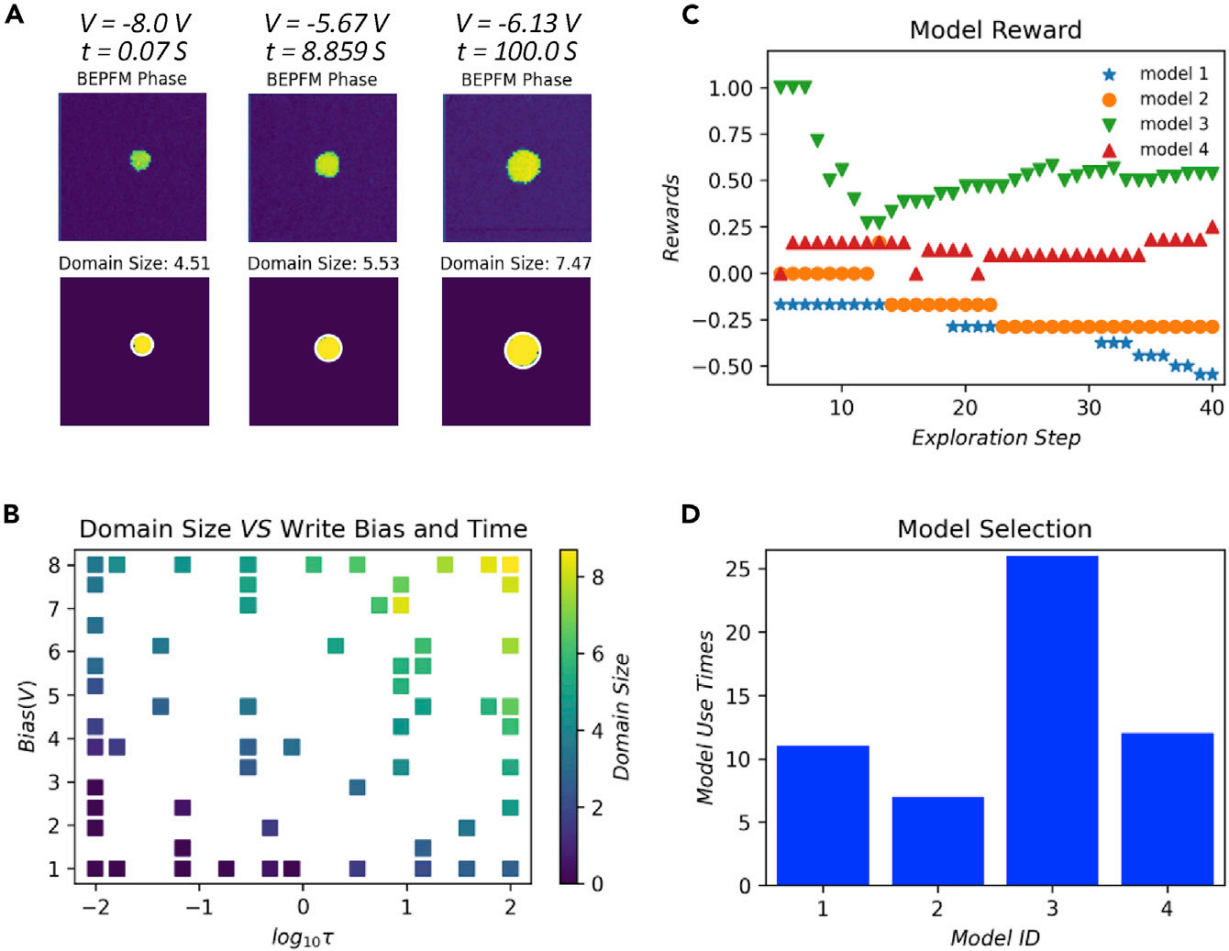
Hypotheses-learning-based automated BEPFM experiment results (A) Three examples of domains written by using different bias and time. The top rows are BEPFM images showing the domains, and the bottom rows are corresponding binary images with domain size detected automatically by the automated workflow. Note that all BEPFM results and domain binary images are shown in Data S2 as a function of the measurement step. (B) Domain size as a function of writing parameters. (C) Model rewards during the hypotheses learning after the initial 5-step warmup phase, during which all models were evaluated at each step. Model III gained a much larger reward than other models, and its reward gradually increased at the latter part of the experiment. (D) Model selection in hypotheses learning, in which model III was selected more often than other models.

Shown in Figure 5 are the hypotheses-learning-based automated BEPFM results. In this experiment, 18 random writing parameters (5% of the writing parameters library) were selected to perform the initial domain writing experiment to provide initial (“seed”) points for hypothesis learning. The obtained domain sizes along with corresponding writing parameters were used as initial training data for the hypothesis-learning algorithm. Then, 40 measurements were performed using writing parameters predicted by the algorithm. Figure 5A shows a few examples of the domains written in the BTO thin film, along with the binary domain images and domain size automatically detected by the workflow. It indicates that in this experiment, both the writing bias and writing time affect the domain size. In Figure 5B, all obtained results are shown as domain sizes as a function of write bias and time. Clearly, the larger bias and longer time result in increased domain sizes. Figure 5C shows the usage times of each model in the 40 step hypotheses learning, where the most often sampled model is model III. Figure 5D shows the evolution of rewards of each model. Model III “won” the most rewards, and its reward values steadily grow in the latter part of the experiment (this explains why it was selected more frequently than other models).

**Figure 6:**
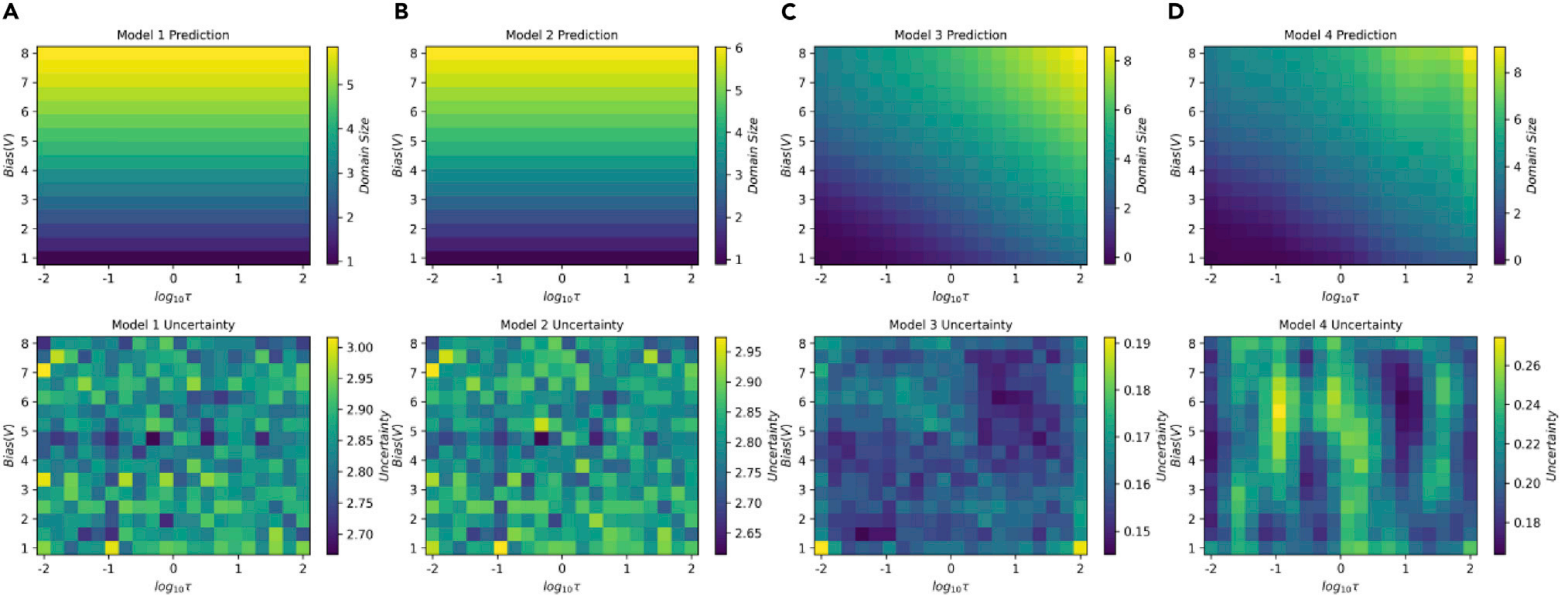
Predictions by all models on the final set of discovered parameters after the competition of the experiment (A–D) Prediction (top row) and corresponding uncertainties (bottom row) by four different models, respectively.

Shown in Figure 6 are predictions and uncertainties by four models after the experiment based on all obtained results. The predictions of models III and IV describe the experimental results (Figure 5B) better, and model III exhibits the lowest uncertainty, indicating that model III, meaning the domain wall pinning on the defect centers, is the correct one. These results suggest that the domain growth in this BTO thin film is determined by the kinetics of the domain wall motion rather than thermodynamics of the screening process, and the effect of surface screen charges is minor. In the automated experiment, the hypothesis learning also actively updates the model parameters. At the competition of the experiment, each model got parameters that best describe the experimental data. The final parameters of each model are summarized in Table 2.

**Table 2:** Final parameters of each model obtained from hypotheses-learning automated experiment

Model	Model equation	Final model parameters								
I	$r\left(V\right)=r_{cr}+r_{0}\sqrt{{\left(\frac{V}{V_{c}}\right)}^{2/3}-1}$	$r_{cr}$			$r_{0}$			$V_{c}$		
		$\bar{x}$	M	s	$\bar{x}$	M	s	$\bar{x}$	M	s
		−0.23	−0.25	0.89	2.35	2.37	0.72	0.53	0.53	0.24
II	$r\left(V\right)=r_{cr}+r_{0}\sqrt[{3}]{{\left(\frac{V}{V_{c}}\right)}^{2}-1}$	$r_{cr}$			$r_{0}$			$V_{c}$		
		$\bar{\bar{x}}$	M	s	$\bar{x}$	M	s	$\bar{x}$	M	s
		0.06	0.84	0.02	1.03	1.03	0.36	0.65	0.59	0.40
III	$r\left(V,t\right)=V^{\alpha }\;\log\;t$	*Α*								
		$\bar{x}$			M			s		
		0.38			0.37			0.05		
IV	$r\left(V,t\right)=V^{\alpha }t^{\beta }$	*Α*			*β*					
		$\bar{x}$	M	s	$\bar{x}$	M	s			
		0.81	0.81	0.01	0.33	0.33	0.00			

(1) *x̅*, mean value; *M*, median value; *s*, SD. (2) All model parameters are obtained from the last time a particular model was sampled in the hypotheses-learning experiment. Model I parameters are obtained from training step 38, measurement step 58; model II parameters are obtained from training step 22, measurement step 42; model III parameters are obtained from training step 40, measurement step 60; model IV parameters are obtained from training step 39, measurement step 59.

### Conclusions

To summarize, we have illustrated the hypothesis-learning-based automated experiment to explore domain switching. The hypothesis list has been built for different domain growth limiting stages, including thermodynamics of domain formation, domain wall pinning, and transport of screening charges at the surfaces. The results indicate that domain growth is ruled by kinetic control.

This approach for probing local bias-induced transformation is general and can be used for other tip-induced reactions and processes, including reversible and irreversible tip-induced electrochemical reactions including electroplating^66^ and nanooxidation.^67^^,^^68^ Note that the detection signal is not limited to the direct measurement of the domain size and can include measured currents, changes in topography, and resonance frequency shifts. As such, it can provide a powerful tool for probing neuromorphic materials^69^ and fuel cell and battery materials,^70^ as well as provide fundamental insights into electrochemical processes on the nanometer scale.^71^

We further note that the hypothesis learning can be broadly used in other automated experiment settings. Currently, this includes exploration of relatively low-dimensional parameter cases for which easy-to-evaluate competing physical models are available such as automated synthesis via microfluidic and robotic systems,^72^ pulsed laser deposition, other forms of materials synthesis, etc.

## Experimental procedures

### Resource availability

#### Lead contact

Further information and requests for resources should be directed to and will be fulfilled by the lead contact, Yongtao Liu, liuy3@ornl.gov.

#### Materials availability

This study did not generate new materials.

### Materials

BTO thin films were grown via pulsed laser deposition (PLD) in 99.9999% pure O_2_ at 700°C. Specifically, first, a 5 nm SrRuO_3_ back electrode was grown on (100) single-sided epitaxial-polished SrTiO_3_ substrates at 100 millitorr with a pulse rate of 5 Hz from a stoichiometric SrRuO_3_ ceramic target. Subsequently, 80 nm BTO was grown at 10 millitorr with a laser pulse rate of 10 Hz from a stoichiometric BTO target. The fluence for both thin-film layers was maintained at approximately 1.2 J/cm^2^. Substrates were prepared by sonication in a warm (∼70°C) deionized water bath for 1 min followed by an anneal at 1,000°C for 12 h to produce TiO_2_ termination with step and terrace surface morphology.

### Automated experiment in PFM

The hypothesis-learning-driven automated BEPFM measurement is based on an Asylum Research Cypher microscope equipped with a National Instruments DAQ card with LabView and a Field Programmable Gate Arrays with Python Jupyter Notebook. For domain writing, FPGA moves the tip to a desired location and applies a DC bias, followed by a BEPFM image measurement performed with NI, FPGA, and Cypher. The FPGA performs scan (move tip) and send to trigger to the NI DAQ card to perform BE measurements simultaneously. These processes are embedded in a Jupyter Notebook. When a measurement finishes, the Jupyter Notebook analyzes the BEPFM phase image to obtain the domain size and saves the domain size in Google Drive. Then, the hypothesis training is performed in Google Colaboratory with this domain size (and previous domain size), followed by saving the next writing parameters for the next experiment.

### Hypothesis learning

The hypothesis learning (hypoAL)^27^ was implemented using the home-build GPax package https://github.com/ziatdinovmax/gpax. The probabilistic models were wrapped into the structured Gaussian processes,^28^ and the BI was performed via the iterative No-U-Turn sampler.^73^ To ensure that wrapped models I and II remained isotropic in time, the kernel length scale for the time dimension was set to a sufficiently large value (1,000), whereas the kernel length scale for the voltage dimension was sampled from a standard weakly informative log-normal prior. For models III and IV, the ARD kernel in both dimensions was sampled from log-normal priors. The acquisition function value in each unmeasured point $x_{*}$ was equal to the posterior predictive uncertainty

$$V\left[f_{*}\right]=\frac{1}{N}\sum_{n=1}^{N}{\left(f_{*}^{n}-\hat{f_{*}}\right)}^{2},with\;\hat{f_{*}}=\frac{1}{N}\sum_{n=1}^{N}P\left(x_{*}|\theta^{n},D\right)\text{,}$$

where $\theta^{n}∼\;P\left(\theta |D\right)$ were samples drawn from the posterior and *D* was the available (measured) data. The reward function was defined as

$$R\left(V_{m}^{i},V_{m}^{i=1}\right)=\left\{ \begin{array}{cc} +1\text{,} & V_{m}^{i}<V_{m}^{i-1}\\ -1\text{,} & V_{m}^{i}\geq V_{m}^{i-1} \end{array}\right.\text{,}$$

where $V_{m}^{i}$ is a median value of posterior predictive uncertainty at step *i*. The python script used to run the hypoAL during the experiment can be found in the supplemental information.

## Data Availability

Code of this study is provided in the supplemental information.

## Supplemental information

Document S1. Appendix A and Note S1

Data S1. Python script used to run the hypoAL during the experiment

Data S2. PFM phase image, binary image showing domain size, domain size as a function of write parameters, model selection, and model reward as a function of measurement step

Document S2. Article plus supplemental information
