# Supplementary material for: Autonomous scanning probe microscopy with hypothesis learning: Exploring the physics of domain switching in ferroelectric materials
Source: Patterns (N Y). 2023 Mar 10;4(3):100704. doi: 10.1016/j.patter.2023.100704 (PMC10028429; doi:10.1016/j.patter.2023.100704)
Supplement: Document S1. Appendix A and Note S1 [file mmc1.pdf]

Patterns, Volume *4*

Supplemental information

Autonomous scanning probe microscopy with hypothesis learning: Exploring the physics of domain switching in ferroelectric materials

Yongtao Liu, Anna N. Morozovska, Eugene A. Eliseev, Kyle P. Kelley, Rama Vasudevan, Maxim Ziatdinov, and Sergei V. Kalinin

Supplemental Experimental Procedures

Autonomous Scanning Probe Microscopy with Hypothesis Learning: Exploring the Physics of Domain Switching in Ferroelectric Materials

Yongtao Liu,[1, a, *] Anna Morozovska,[2] Eugene Eliseev,[2,3] Kyle P. Kelley,[1] Rama Vasudevan,[1] Maxim Ziatdinov,[1,4,b] and Sergei V. Kalinin[1, c]

1 Center for Nanophase Materials Sciences, Oak Ridge National Laboratory, Oak Ridge, TN 37922, United States

2 Institute of Physics, National Academy of Sciences of Ukraine, 46, pr. Nauky, 03028 Kyiv, Ukraine

3 Institute for Problems of Materials Science, National Academy of Sciences of Ukraine, Krjijanovskogo 3, 03142 Kyiv, Ukraine

4 Computational Sciences and Engineering Division, Oak Ridge National Laboratory, Oak Ridge, TN 37831, United States

[a, b, c] Corresponding authors' email addresses: liuy3@ornl.gov ; ziatdinovma@ornl.gov ; sergei2@utk.edu

*Lead Contact's email address: liuy3@ornl.gov

1. **Supplementary Appendix A.** Models of domain growth
2. **Supplementary Note.** Challenges to Implementing Machine Learning-Driven Autonomous Microscopy
3. **Data S1.** Python script used to run the hypoAL during the experiment
4. **Data S2.** PFM phase image, binary image showing domain size, domain size as a function of write parameters, model selection, and model reward as a function of measurement step.

1. Appendix A. Models of domain growth

A. Thermodynamic models 1-3

The voltage dependences of the equilibrium domain sizes are listed in **Table I**. They were selected based on Refs. [i, ii, iii].

Table I. Adapted from Ref. [i]

**Domain charac-teristics**	**Intrinsic model of domain formation for thick films ($h >> \gamma d$)**		
	**Case (i)**: the complete screening of depolarization field	**Case (ii)**: no screening of depolarization field, but the wall can be thick	**Case (iii):** LM- model infinitely thin domain walls
Coercive bias V_c	$V_c = \gamma d \cdot E_c$	$V_c \approx \gamma d(E_c + \Delta E)$,<br>$\Delta E = 2P_S/(\varepsilon_0 \varepsilon_{11} \gamma^2)$	Analytical expression for V_c is thickness-dependent
Domain onset at $V \to V_c$	Onset is the second order phase transition, i.e., activationless.<br>An oblate domain appears with very small sizes $r(V_c) = 0$ and $l(V_c) \sim r^2(V_c)$.	Onset is the first order phase transition, i.e., the barrier should exist.<br>Nucleus is very prolate and spike-like $r(V_c) \ll l(V_c)$, and rapidly growth downward.	Activation barrier exists for the length, since prolate stable domain appears with nonzero sizes $l(V_c) > r(V_c) > 0$
Sizes vs. bias V, at $V \geq V_c$	$r(V) = d\sqrt{\left(\frac{V}{V_c}\right)^{2/3} - 1}$<br>$l(V) = \gamma \cdot d\sqrt{\frac{V}{V_c} - 1}$	$r(V) \approx r_{cr} + d\sqrt{\left(\frac{V}{V_c}\right)^{2/3} - 1}$<br>$l(V) = l_{cr} + l_0\sqrt{\frac{V}{V_c} - 1}$<br>length $l >> r$ is determined by depolarization energy value.	$r(V) = r_{cr} + r_0\sqrt[3]{\left(\frac{V}{V_c}\right)^2 - 1}$<br>$l(V) = l_{cr} + l_0\sqrt{\left(\frac{V}{V_c}\right)^2 - 1}$<br>length $l >> r$ is determined by the interplay between the depolarization energy and domain wall surface energy.
Sizes vs. bias V, at $V \gg V_c$	$r(V) \sim V^{1/3}$<br>$l(V) \sim V^{1/2}$<br>$r^3/l^2 \approx \gamma^{-2}$	$r(V) \sim V^{1/3}$<br>$l(V) \sim V^{1/2}$<br>$r^3/l^2 \approx \gamma^{-2}$	$r(V) \sim V^{2/3}$<br>$l(V) \sim V$<br>$r^3/l^2 \approx \text{const}$

γ is a dielectric anisotropy factor

B. Kinetic model 4

The domain wall velocity in a ferroelectric containing defects is related to an acting electric field E as [iv, v]

$$v(r) \approx v_0 \, exp\left[-\left(\frac{E_{th}}{E}\right)^{\mu}\right] \qquad (1a)$$

Where μ is a positive number, so-called exponential factor, that is typically close to unity. E_{th} is the activation field, defined as the threshold field, above which the domain wall is unpinned

by defects. Another situation corresponds to a rapid wall motion (in very high fields) without pinning effects:

$$v(r) \approx v_0 \left(\frac{E}{E_{cr}}\right)^{\chi}, \qquad E \gg E_{cr} \tag{1b}$$

Using the simplest form for a normal component of the tip field,

$$E_z(r,0) = \frac{Vd^2}{\gamma(r^2+d^2)^{3/2}}, \tag{2}$$

and ignore the depolarization field in Eq.(1a), we obtain:

$$v(r) \approx v_0\, exp\left[-\left(\frac{\gamma E_{th}}{Vd^2}\sqrt{(r^2+d^2)^3}\right)^{\mu}\right] \tag{3a}$$

Here γ is a dielectric anisotropy factor, r is a surface distance from the tip axis, and V is the bias applied between the tip and the bottom electrode. So, the velocity at the domain boundary depends on the domain radius. Note that one can neglect the depolarization field under specific conditions, such as the lateral growth of uncharged domain wall, or arbitrary growth accompanied by a local screening of depolarization field by free carriers. However, the screening carriers should also affect on the tip field, so the second case is exotic.

For the case $E \gg E_{cr}$

$$v(r) \approx v_0 \left(\frac{Vd^2}{\gamma E_{cr}(r^2+d^2)^{3/2}}\right)^{\chi}, \tag{3b}$$

Where the factor χ is positive.

The instant domain radius is $r(t) = \int_0^t v(t')dt'$, since $\frac{dr}{dt} = v$. So, to define the domain wall velocity we need to solve a differential equation:

$$\frac{dr}{dt} \approx v_0\, exp\left[-\left(\frac{\gamma E_{th}}{Vd^2}\sqrt{(r^2+d^2)^3}\right)^{\mu}\right], \tag{4a}$$

or, for the case $E \gg E_{cr}$,

$$\frac{dr}{dt} \approx v_0 \left(\frac{Vd^2}{\gamma E_{cr}(r^2+d^2)^{3/2}}\right)^{\chi}, \tag{4b}$$

Eq.(4a) has no simple analytical solution. Putting $\mu = 1/3$ and regarding that we are interested in the case $r \gg d$, we obtain that $v_0 t = \left(\frac{Vd^2}{\gamma E_{th}}\right)^{1/3}\left(exp\left[\left(\frac{\gamma E_{th}}{Vd^2}\right)^{1/3} r\right] - 1\right)$. So, the approximate solution is

$$r(t) \approx \left(\frac{Vd^2}{\gamma E_{th}}\right)^{1/3} ln\left[1 + \left(\frac{\gamma E_{th}}{Vd^2}\right)^{1/3} v_0 t\right]. \tag{5a}$$

Expression (5a) describes a slow logarithmic creep of the domain wall, at that $r(V) \sim V^{1/3}$ at high voltages. Actually at $\left(\frac{\gamma E_{th}}{Vd^2}\right)^{1/3} v_0 t \gg 1$, we obtain that $r(t) \sim \left(\frac{Vd^2}{\gamma E_{th}}\right)^{1/3} ln[v_0 t]$. However,

the lateral growth stops at equilibrium domain sizes after the pulse ending, which can be calculated from thermodynamic description.

Eq.(4b) has a simple analytical solution. Being interested in the case $r \gg d$, we can obtain different power laws:

$$r(t) \approx \frac{1}{3\chi+1}\left[v_0 t\left(\frac{V}{\gamma E_{cr} d}\right)^{\chi}\right]^{\frac{1}{3\chi+1}} \sim V^{\frac{\chi}{3\chi+1}}(v_0 t)^{\frac{1}{3\chi+1}}. \qquad (5b)$$

Note that the opposite case $r \ll d$ seems to be unphysical, since it means very small times. However, Eq.(5b) states that at positive χ, the domain growth is sub-linear both in voltage and it time.

The intrinsic coercive field E_c is well-known, and can be expressed via the LGD functional expansion coefficients as [vi]:

$$E_c = \begin{cases} \frac{2}{3\sqrt{3}}\sqrt{-\frac{\alpha^3}{\beta}} \text{ ,for second order ferroelectrics,} \\ \frac{2}{5}\left(2\beta + \sqrt{9\beta^2 - 20\alpha\delta}\right)\left(\frac{2\alpha}{-3\beta-\sqrt{9\beta^2-20\alpha\delta}}\right)^{3/2} \text{ for first order ferroelectrics.} \end{cases} \qquad (6)$$

Note that expressions listed in Table I for the case of the electric excitation by the localized probe field with characteristic scale d differs from the semi-empirical Kay-Dunn law [vii], which stated that $r \sim h^{2/3}$and coercive field $E_{cr} \sim h^{-2/3}$ for homogeneous external field.

2. **Supplementary Note. Challenges to Implementing Machine Learning-Driven Autonomous Microscopy**

Nowadays, most commercialized SPM microscopes do not allow programmatic control (e.g. control by Python program), so the first challenge is to enable the automation of microscopes. This requires lots of effort to develop electronics and software in order to control the microscope through Python program. In our work, we used an FPGA and developed a script that allows us to send voltage to (1) the piezo scanner of the microscope, and (2) the tip; so that we can drive the tip to perform PFM scan and apply a voltage to the tip to write a domain. Second, multiple software and Python programs are used for this experiment, these software and programs need different operating systems. For instance, in this experiment, hypothesis learning requires a Linux environment but other software requires a Windows environment. Thus, in order to perform real-time hypothesis learning during measurement, we need to transfer experiment results and hypothesis learning analysis results between Linux and Windows computers in real-time. Here, we used an ethernet cable to enable real-time data transfer. However, we would like to note that these challenges and corresponding solutions may vary in different labs, depending on microscopes and lab conditions.

References

[i] A.N. Morozovska, E.A. Eliseev, Yulan Li, S.V. Svechnikov, P. Maksymovych, V.Y. Shur, Venkatraman Gopalan, Long-Qing Chen, and S.V. Kalinin. Thermodynamics of nanodomain formation and breakdown in Scanning Probe Microscopy: Landau-Ginzburg-Devonshire approach. Phys. Rev. B. 80, 214110 (2009).

[ii] Jason Chen, Alexei Gruverman, Anna N. Morozovska and Nagarajan Valanoor. Sub-Critical Field Domain Reversal in Epitaxial Ferroelectric Films. J. Appl. Phys. **116**, 124109 (2014); http://dx.doi.org/10.1063/1.4896730

[iii] N. Panwar, I. K. Bdikin, A.N. Morozovska, and A. L. Kholkin, J. Appl. Phys **112**, 052019 (2012)

[iv] M. Molotskii, J. Appl. Phys. **97**, 6234 (2005).

[v] P. Paruch, T. Giamarchi, T. Tybell, and J. M. Triscone, J. Appl. Phys. **100**, 051608 (2006).

[vi] S. Ducharme, V. M. Fridkin, A.V. Bune, S. P. Palto, L. M. Blinov, N. N. Petukhova, S. G. Yudin, Phys. Rev. Lett. **84**, 175 (2000).

[vii] H.F. Kay and J.W. Dunn, Phil. Mag. **7**, 2027 (1962).
