# Supplementary material for: Autonomous scanning probe microscopy with hypothesis learning: Exploring the physics of domain switching in ferroelectric materials
Source: Patterns (N Y). 2023 Mar 10;4(3):100704. doi: 10.1016/j.patter.2023.100704 (PMC10028429; doi:10.1016/j.patter.2023.100704)
Supplement: Data S1. Python script used to run the hypoAL during the experiment [file mmc2.pdf]

```python
import argparse
import os
from typing import Callable, Dict, List, Tuple, Type, Union

import gpax
import jax.numpy as jnp
import numpy as onp
import numpyro

import matplotlib.pyplot as plt

gpax.utils.enable_x64()

### Set basic parameters ###
EPS = 0.4  # epsilon in epsilon-greedy policy
ACQ = gpax.acquisition.UE  # acquisition fucntion for active learning
SAVEPATH = "./"  # Path for stroring current reward records (use Google Drive if running in Colab)
WARMUP_STEPS = 5  # number of steps in the warmup phase (not to be confused with the MCMC warmup)
NOISE = 10  # noise level defined as s in LogNormal(0, s)
MCMC_WARMUP = 5000  # Number of MCMC warmup samples
MCMC_SAMPLES = 5000  # Number of MCMC samples
MCMC_CHAINS = 1  # Number of MCMC chains

### Define possible models of system's behavior as deterministic functions ###

def model2(x: jnp.ndarray, params: Dict[str, float]) -> jnp.ndarray:
    """
    r = r_c + d * ((V/V_c)^{2/3} - 1)^{1/2}
    """
    return params["r_c"] + params["r_0"] * jnp.sqrt((x[:, 0] / params["V_c"])**(2/3) - 1)


def model3(x: jnp.ndarray, params: Dict[str, float]) -> jnp.ndarray:
    """
    r = r_c + r_0 * ((V/V_c)^2 - 1)^{1/3}
    """
    return params["r_c"] + params["r_0"] * jnp.cbrt((x[:, 0] / params["V_c"])**2 - 1)


def model4(x: jnp.ndarray, params: Dict[str, float]) -> jnp.ndarray:
    """
    r = V^{beta} * log(tau)
    """
    return x[:, 0]**params["beta"] * x[:, 1]


def model5(x: jnp.ndarray, params: Dict[str, float]) -> jnp.ndarray:
    """
    r = V^{alpha} * tau^{beta}
    """
    tau = 10**x[:, 1]  # 10^{log_{10}(tau)}
    return x[:, 0]**params["alpha"] * tau**params["beta"]

### Put priors over parameters of each model (to make them probabilistic) ###
```

```python

def model2_priors() -> Dict[str, jnp.ndarray]:
    r_c = numpyro.sample("r_c", numpyro.distributions.Normal(0, 1))
    r_0 = numpyro.sample("r_0", numpyro.distributions.LogNormal(0, 1))
    V_c = numpyro.sample("V_c", numpyro.distributions.LogNormal(0, 1))
    return {"r_c": r_c, "r_0": r_0, "V_c": V_c}


def model3_priors() -> Dict[str, jnp.ndarray]:
    r_c = numpyro.sample("r_c", numpyro.distributions.Normal(0, 1))
    r_0 = numpyro.sample("r_0", numpyro.distributions.LogNormal(0, 1))
    V_c = numpyro.sample("V_c", numpyro.distributions.LogNormal(0, 1))
    return {"r_c": r_c, "r_0": r_0, "V_c": V_c}


def model4_priors() -> Dict[str, jnp.ndarray]:
    beta = numpyro.sample("beta", numpyro.distributions.Uniform(0.33, 1.2))
    return {"beta": beta}


def model5_priors() -> Dict[str, jnp.ndarray]:
    alpha = numpyro.sample("alpha", numpyro.distributions.Uniform(0.8, 1.2))
    beta = numpyro.sample("beta", numpyro.distributions.Uniform(0.33, 1.2))
    return {"alpha": alpha, "beta": beta}


### Custom kernel prior ###

def kernel_prior() -> Dict[str, jnp.ndarray]:
    k_length1 = numpyro.sample("k_length1", numpyro.distributions.LogNormal(0, 1))
    k_length2 = numpyro.deterministic("k_length2", jnp.array(1000))
    k_length = numpyro.deterministic("k_length", jnp.array([k_length1, k_length2]))
    k_scale = numpyro.sample("k_scale", numpyro.distributions.LogNormal(0, 1))
    return {"k_length": k_length, "k_scale": k_scale}

### Utility functions for active learning ###

def get_best_model(record: Union[onp.ndarray, jnp.ndarray]) -> int:
    return record[:, 1].argmax()


def update_record(record: onp.ndarray, action: int, r: float) -> onp.ndarray:
    new_r = (record[action, 0] * record[action, 1] + r) / (record[action, 0] + 1)
    record[action, 0] += 1
    record[action, 1] = new_r
    return record


def get_reward(obj_history: List[float],
               obj: Union[onp.ndarray, jnp.ndarray]) -> int:
    """A reward of +/-1 is given if the integral uncertainty at the current step
    is smaller/larger than the integral uncertainty at the previous step"""
    if jnp.nanmedian(obj) < obj_history[-1]:
        r = 1
    else:
        r = -1
    return r
```

```python


def step(model: Callable[[jnp.ndarray, Dict[str, jnp.ndarray]], jnp.ndarray],
         model_prior: Callable[[], Dict[str, jnp.ndarray]],
         kernel_prior: Callable[[], Dict[str, jnp.ndarray]],
         X_train: jnp.ndarray, y_train: jnp.ndarray, X_new: jnp.ndarray,
         acq_fn: Callable[[jnp.ndarray, Type[gpax.ExactGP], jnp.ndarray], jnp.ndarray],
         num_restarts: int = 1) -> Tuple[jnp.ndarray, Type[gpax.ExactGP]]:
    """Compute model posterior and use it to derive acqusition function"""
    sgr = numpyro.diagnostics.split_gelman_rubin
    for i in range(num_restarts):
        rng_key, rng_key_predict = gpax.utils.get_keys(i)
        # Get/update model posterior
        gp_model = gpax.ExactGP(
            2, 'Matern', model, kernel_prior, model_prior,
            noise_prior=lambda: numpyro.sample("noise", numpyro.distributions.LogNormal(0, NOISE)))
        gp_model.fit(
            rng_key, X_train, y_train, num_warmup=MCMC_WARMUP,
            num_samples=MCMC_SAMPLES, num_chains=MCMC_CHAINS)
        rhats = [sgr(v).max() for v in gp_model.get_samples(1).values()]
        if max(rhats) < 1.2:
            break
    # Compute acquisition function
    obj = acq_fn(rng_key_predict, gp_model, X_new)
    return obj, gp_model


def load_dataset(filepath: str) -> Tuple[jnp.ndarray]:
    """Load observed data, unobserved points, and history of rewards"""
    dataset = onp.load(filepath)
    X_measured = dataset["X_measured"]
    y_measured = dataset["y_measured"]
    indices_measured = dataset["indices_measured"]
    X_unmeasured = dataset["X_unmeasured"]
    indices_unmeasured = dataset["indices_unmeasured"]
    return (X_measured, y_measured, indices_measured, X_unmeasured, indices_unmeasured)


def load_records(n_models) -> Tuple[onp.ndarray, List[float]]:
    if not os.path.exists(os.path.join(SAVEPATH, "history.npz")):
        record = onp.zeros((n_models, 2))
        obj_history = []
    else:
        history = onp.load("history.npz")
        record = history["record"]
        obj_history = history["obj_history"].tolist()
    return record, obj_history


def main(args):
    # Make a list of models and corresponding model priors
    models = [model2, model3, model4, model5]
    model_priors = [model2_priors, model3_priors, model4_priors, model5_priors]
    assert len(models) == len(model_priors)
    n_models = len(models)
    # Load data
```

```
    (X_measured, y_measured, indices_measured,
     X_unmeasured, indices_unmeasured) = load_dataset(args.filepath)
    # Load history
    record, obj_history = load_records(n_models)
    # Run warmup phase for the first 3 exploration steps
    warmup = jnp.clip(WARMUP_STEPS - len(obj_history), 0)
    if warmup:  # warmup phase
        print("Warmup step {}".format(len(obj_history) + 1))
        obj_median_all, obj_all = [], []
        for i, model in enumerate(models):
            k_prior = kernel_prior if i < 2 else None
            obj, _ = step(model, model_priors[i], k_prior,
                          X_measured, y_measured, X_unmeasured, ACQ)
            record[i, 0] += 1
            obj_all.append(obj)
            obj_median_all.append(jnp.nanmedian(obj).item())
        # Reward a model that has the smallest integral/median uncertainty
        idx = onp.argmin(obj_median_all)
        # Get the uncertainty map for the rewarded model
        obj = obj_all[idx]
        # Update records
        record[idx, 1] += 1
        obj_history.append(obj_median_all[idx])

        if WARMUP_STEPS == len(obj_history):
            record[:, 1] = record[:, 1] / WARMUP_STEPS
    else:  # epsilon-greedy exploration

        if onp.random.random() > EPS:
            idx = get_best_model(record)
        else:
            idx = onp.random.randint(len(models))
        print("Using model {}".format(idx+1))
        # Derive acqusition function with the selected model
        k_prior = kernel_prior if idx < 2 else None
        obj, _ = step(models[idx], model_priors[idx], k_prior,
                      X_measured, y_measured, X_unmeasured, ACQ, 2)
        # Get reward
        r = get_reward(obj_history, obj)
        # Update records
        record = update_record(record, idx, r)
        obj_history.append(jnp.nanmedian(obj).item())


    # Compute the next measurement point
    next_point_idx = obj.argmax()

    # Save suggested point and model idx
    _path = os.path.join(SAVEPATH, "records_misc.npz")
    if not os.path.exists(_path):
        onp.savez(_path, points=next_point_idx, model_ids=idx)
    else:
        records_misc = onp.load(_path)
        points = records_misc["points"]
        points = onp.append(points, next_point_idx)
        model_ids = records_misc["model_ids"]
        model_ids = onp.append(model_ids, idx)
```

```
        onp.savez(_path, points=points, model_ids=model_ids)
    # save records of rewards and uncertainty
    onp.savez(os.path.join(SAVEPATH, "history.npz"), record=record, obj_history=obj_history

    # Display the current model rewards
    print("\nCURRENT MODEL REWARDS")
    for i, r in enumerate(record):
        print("model {}:  counts {}  reward (avg) {}".format(i+1, (int(r[0])),
onp.round(r[1], 3)))
    # Display tjhe suggested point
    print("\nNEXT POINT ID: {}, NEXT POINT VALUE: {}".format(
                next_point_idx, X_unmeasured[next_point_idx]))
    onp.save(os.path.join(SAVEPATH, "next_idx.npy"), next_point_idx)

if __name__ == "__main__":

    parser = argparse.ArgumentParser()
    parser.add_argument("filepath", nargs="?", type=str)
    args = parser.parse_args()
    main(args)
```
