# Supplementary material for: Autonomous scanning probe microscopy with hypothesis learning: Exploring the physics of domain switching in ferroelectric materials
Source: Patterns (N Y). 2023 Mar 10;4(3):100704. doi: 10.1016/j.patter.2023.100704 (PMC10028429; doi:10.1016/j.patter.2023.100704)
Supplement: Document S2. Article plus supplemental information [file mmc4.pdf]

Patterns

Article

Autonomous scanning probe microscopy with hypothesis learning: Exploring the physics of domain switching in ferroelectric materials

Graphical abstract

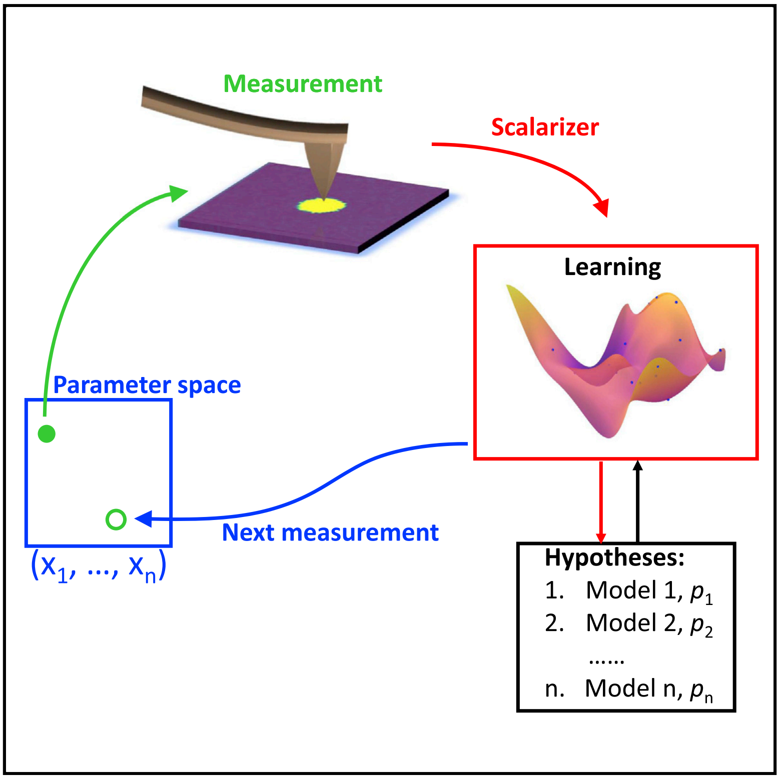

Highlights

- Hypothesis learning combines machine learning with physical hypotheses
- Hypothesis learning enables physical discovery in the experiment
- Hypothesis learning is implemented in operating scanning probe microscopy
- Hypothesis learning identifies the ferroelectric domain growth mechanism autonomously

Authors

Yongtao Liu, Anna N. Morozovska, Eugene A. Eliseev, Kyle P. Kelley, Rama Vasudevan, Maxim Ziatdinov, Sergei V. Kalinin

Correspondence

liuy3@ornl.gov (Y.L.),
ziatdinovma@ornl.gov (M.Z.),
sergei2@utk.edu (S.V.K.)

In brief

Hypothesis-learning-driven automated scanning probe microscopy allows physical discovery during experiments, where the hypothesis learning algorithm implemented on an operating microscope identifies the physical mechanism behind the material's response.

Liu et al., 2023, Patterns *4*, 100704
March 10, 2023 © 2023 The Author(s).
https://doi.org/10.1016/j.patter.2023.100704

CellPress

Patterns

CellPress
OPEN ACCESS

Article

Autonomous scanning probe microscopy with hypothesis learning: Exploring the physics of domain switching in ferroelectric materials

Yongtao Liu,[1,5,*] Anna N. Morozovska,[2] Eugene A. Eliseev,[2,3] Kyle P. Kelley,[1] Rama Vasudevan,[1] Maxim Ziatdinov,[1,4,*] and Sergei V. Kalinin[1,*]
[1]Center for Nanophase Materials Sciences, Oak Ridge National Laboratory, Oak Ridge, TN 37922, USA
[2]Institute of Physics, National Academy of Sciences of Ukraine, 46, pr. Nauky, 03028 Kyiv, Ukraine
[3]Institute for Problems of Materials Science, National Academy of Sciences of Ukraine, Krjijanovskogo 3, 03142 Kyiv, Ukraine
[4]Computational Sciences and Engineering Division, Oak Ridge National Laboratory, Oak Ridge, TN 37831, USA
[5]Lead contact
*Correspondence: liuy3@ornl.gov (Y.L.), ziatdinovma@ornl.gov (M.Z.), sergei2@utk.edu (S.V.K.)
https://doi.org/10.1016/j.patter.2023.100704

THE BIGGER PICTURE The need for automated experimentation has been recently recognized across multiple areas of instrument-based sciences, including X-ray scattering, electron, and scanning probe microscopy, necessitating the development of algorithms for navigating multidimensional compositional or processing spaces. Autonomous discovery of physical laws via experiment has emerged as a rapidly emerging topic for machine learning in physical sciences. Here, we report the development of a hypothesis-learning-based automated experiment, which autonomously identifies the mechanism behind materials' response during operating experiments. This approach can be applied to a broad range of physical and chemical experiments with relatively low-dimensional control parameter space and for which the possible competing models of the system behavior that ideally cover the full range of physical eventualities are known.

1 2 3 4 5 **Proof-of-Concept:** Data science output has been formulated, implemented, and tested for one domain/problem

SUMMARY

Using hypothesis-learning-driven automated scanning probe microscopy (SPM), we explore the bias-induced transformations that underpin the functionality of broad classes of devices and materials from batteries and memristors to ferroelectrics and antiferroelectrics. Optimization and design of these materials require probing the mechanisms of these transformations on the nanometer scale as a function of a broad range of control parameters, leading to experimentally intractable scenarios. Meanwhile, often these behaviors are understood within potentially competing theoretical hypotheses. Here, we develop a hypothesis list covering possible limiting scenarios for domain growth in ferroelectric materials, including thermodynamic, domain-wall pinning, and screening limited. The hypothesis-driven SPM autonomously identifies the mechanisms of bias-induced domain switching, and the results indicate that domain growth is ruled by kinetic control. We note that the hypothesis learning can be broadly used in other automated experiment settings.

INTRODUCTION

Machine learning (ML) methods are rapidly becoming an inseparable part of physical sciences, with the applications ranging from astronomy[1,2] and high energy physics[3] to materials science[4–6] and microscopy.[7] While early demonstrations of ML have been shown in a variety of domain disciplines including computational sciences and first-principles theory,[8,9] microscopy,[10] materials growth,[11] theory-experiment matching,[12] and many others, these efforts remained largely isolated. In theory and computation, the watershed moment was the publication of the seminal 2006 paper by Ceder[13] that launched the materials genome project and subsequently gave rise to multiple ML efforts. The second key point was the demonstration of

This is an open access article under the CC BY license (http://creativecommons.org/licenses/by/4.0/).

deep learning by Krizhevsky[14] that ushered in the deep-learning era of today and sparked enthusiasm and investment toward ML applications across multiple disciplines. Currently, the most effort in the ML field is concentrated on classical big data approaches. Applications such as GPT and DALL-E have captured the attention of professionals and the general community.[15,16]

However, despite their power for image analysis and natural language applications, these big data methods are remarkably limited in domain applications, where the volumes of data sufficient to sample the distribution are rare. In the correlative models, complications arise due to the out-of-distribution shift effects, which are explored both in model examples and in applications in medical imaging and automated driving. Secondly, compared with classical ML problems, physical sciences offer a challenge of active learning with very limited experimental budget. However, it is also well understood that in physical sciences, the nature of scientific domain offers a rich set of prior knowledge in forms ranging from known physical laws and constraints and expected parameter values to difficult-to-define physical intuition.

Particularly of interest are the applications of active learning where the ML agent interacts with a physical object via a suitable measurement tool, i.e., an automated experiment. The need for automated experimentation has been recently recognized across multiple areas of instrument-based sciences, including X-ray scattering, electron, and scanning probe microscopy.[17–19] Similarly, rapid growth in automated synthesis platforms, including computer-controlled synthesis,[20] fully automated labs,[21] microfluidic systems,[22] and combined human-high-throughput experimentation workflows,[23] necessitates the development of algorithms for navigating multidimensional compositional or processing spaces. Notably, the (initial) requirements for automated experimentation in microscopy and synthesis are close and allow for the use of classes of algorithms based on Bayesian optimization (BO).[24–26]

The important limitation of the classical BO strategies with the Gaussian process is the use of the non-parametric kernel-based models. In this case, the internal correlations across the data space are used to select the locations for a new experiment. However, these models do not contain any specific physical assumptions or relationships. Hence, in many cases, the efficiency of BO-based methods is only within an order of magnitude from classical grid search-based strategies. At the same time, often, the behaviors of these systems are understood within potentially competing theoretical models or hypotheses.

Recently, we have introduced the approach for physics-informed BO in automated experiments, referred to as hypothesis learning.[27] In this approach, a list of possible models (hypotheses) of the system behavior is established prior to the automated experiment. The hypotheses in this case are the analytical expressions (or other fast computational schemes), with partial knowledge of the associated parameters in the form of Bayesian priors formed based on the analysis of physics of observed phenomena. During the experiment, the algorithm aims to narrow down the range of possible hypotheses following a certain optimization policy, i.e., it tries to establish the best model of the system's behavior within the smallest number of steps. The thus identified model will represent the mechanism of the observed physical phenomena. Ideally, the list of hypotheses will enumerate possible scenarios for materials behavior; however, if the correct model is not a part of the list, the algorithm reverts to the structured model closest to the ground-truth behavior or adopts a structureless Gaussian prior. In this manner, the algorithm discovers the physical laws operating in the studied system.

Here, we illustrate the hypothesis-learning-based automated experiment for the explorations of the domain-switching mechanisms in classical ferroelectric materials. While shown for a model system, this approach is more general and can be broadly used for exploration of other scanning probe microscopy (SPM)-based electrochemical reactions and for automated experimentation in scattering, microscopy, and materials synthesis.

RESULTS AND DISCUSSION

Principles of hypothesis learning

The bedrock element of physical sciences is the validated set of quantitative symbolic relationships between the physical parameters, numerical constants, and observables. Examples range from the fundamental laws of the Newtonian mechanics to expressions defining current-voltage relationships in semiconductors. In certain cases, these relationships are derived from fundamental laws and symmetries. In others, they represent a useful empirical generalization, valid under specific conditions. Using these relationships underpins all areas of physics research, and deriving these relationships is often equated with the understanding of the relevant physical mechanisms.

The research process often involves iterative cycles between the acquisition of the experimental data and their interpretation in terms of specific models. In some cases, the underlying symbolic relationships are discovered via exploratory data analysis, with the subsequent interpretation based on the functional form of derived relationships. In other cases, a number of competing models can be derived based on prior knowledge and fundamental physical laws, and the model best matching the experiment is selected to represent the relevant physics. In all cases, models include not only the symbolic form, per se, but also the expected values of the internal parameters that are known with different degree of certainty, naturally cast in the Bayesian inference (BI) framework.

Hypothesis learning is developed as an approach to implement this iterative cycle as a part of the automated experiment (Figure 1).[27] Here, the automated experiment generally refers to sequential (or batch) measurements of a target functionality over pre-defined parameter space. The scalarizer function reduces the (potentially vector-valued) functionality to a single scalar, defined to represent a measure of the experimentalist's interest in a specific physical property or response. Several possible hypotheses describing the system's behavior are available to complement an automated experiment. The hypothesis generally refers to a model predicting the functionality of interest (or its scalarized form) over the parameter space. Ideally, the list of models reflects the full list of possible mechanisms active within the material and fully covers the possible physical scenarios. The key requirement of the model is the ease of calculation, as is necessary to perform Bayesian evaluations based on Markov chain Monte Carlo techniques. Here, for convenience, we use the hypotheses in the symbolic equation form; however, this requirement can be relaxed to numerical models. The

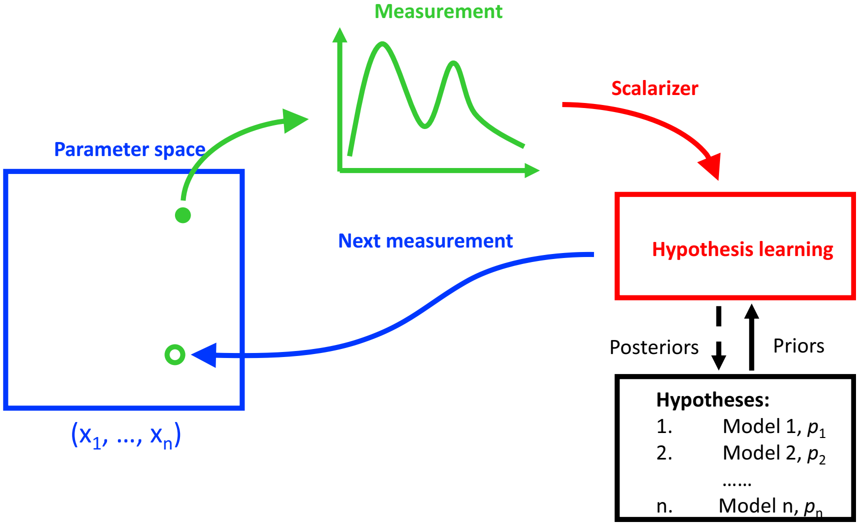

Figure 1. Schematics of the hypothesis learning in automated experiment

The measurement is performed in a selected location(s) in the parameter space. These can be control parameters of experiment, concentrations in the phase diagram, or image plane. The measurement result is converted to a scalar measure of interest. The hypothesis-learning agent uses the measurement to establish the posterior probabilities of a sampled model and select next location(s) in parameter space for measurements. Note that models corresponding to different physical scenarios (hypotheses) return the scalar values derived via the same scalarizer as the experiment.

symbolic expression and associated prior distributions of parameters define a single hypothesis. A list of possible hypotheses with associated prior probabilities (which may or may not include the ground truth one) is a second component of hypothesis learning.

During the hypothesis learning, the agent performs the experiment, returning the scalarized value of the functionality of interest in a selected point of the parameter space. The measured values are used to perform the BI on the list of probabilistic models (hypotheses) wrapped into the structured Gaussian processes,[28] generating posterior probabilities of the models' parameters. The latter is used to obtain posterior predictive uncertainties over the unmeasured points of the parameters space. The model that produced the lowest predictive uncertainty is assigned a positive reward value and is used to sample the next measurement point according to a pre-defined acquisition function. Because running BI for every model on the list at each step is computationally expensive, we do it only for several steps ("warm-up phase") and then switch to the epsilon-greedy policy for sampling a single model (hypothesis) at each step. If the sampled model reduces/increases the predictive uncertainty compared with the previous step, it receives a positive/negative reward.

Mechanisms for ferroelectric domain growth

The phenomenological mechanisms for ferroelectric domain growth in piezoresponse force microscopy (PFM) have been extensively explored for over two decades.[29–31] The early studies have established the phenomenological relationship between the size of the formed domain and the parameters of the bias pulse applied to the tip.[32–35] The initial theoretical analyses of the domain switching were based on either purely thermodynamic considerations in the rigid ferroelectric,[36–38] Ginzburg-Landau approximations,[39,40] or analysis of the domain wall motion in the electrostatic field of the probe.[35,41,42] At the same time, these analyses have demonstrated that thermodynamics of polarization switching strongly depends on the effectiveness of the screening process on the top surface. Correspondingly, the kinetics of the process can be limited by the screening process rather than intrinsic material behavior. From the experimental perspective, ample evidence exists toward the role of screening charge dynamics in switching through observations of charge injection,[43,44] back switching and formation of bubble domains,[45–49] chaotic switching dynamics[50] and formation of complex domains,[51] vortices, and skyrmions.[52–54]

Hence, despite its apparent simplicity, domain switching in ferroelectrics is a complex process that is affected by the intrinsic thermodynamics of the domain formation, domain wall pinning in the spatially non-uniform probe field, and screening charge generation and dynamics. Any of these can serve as a process limiting stage, forming an ideal setting for hypothesis-learning applications. Importantly, at the mesoscopic level, these mechanisms provide the full range of possible physical eventualities, and thus the hypothesis list is assumed to be complete.

Here, we enumerate the hypotheses for the domain growth based on (1) thermodynamic control in the presence or absence of surface screening charges, (2) kinetic control of domain wall motion via pinning, or (3) kinetic control via screening charge dynamics. This list of possible limiting factors is exhaustive for the domain formation in PFM, and hence the selected hypotheses are expected to cover the full list of experimental eventualities. The analysis of the laws of domain growth in these cases was carried out by a number of groups over the last two decades,[34,55–59] and below we give only corresponding approximate expressions. The detailed voltage dependences of the equilibrium domain sizes (1–3) are described in details in Appendix A. Figure 2 illus\eqalign{trates schematically the domain nucleation models.

Here, model I corresponds to the partial internal screening of the depolarization field by space charge carriers and domain wall thickening. For this case, the equilibrium domain radius depends on the tip voltage as

$$r(V) \approx r_{cr} + d\sqrt{\left(\frac{V}{V_C}\right)^{2/3} - 1}, \qquad \text{(Equation 1)}$$

meaning a jump at V_c corresponding to the first-order phase transition and further growth as roughly as $(V/V_c)^{1/3}$. For complete screening, the jump disappears, and the equilibrium domain radius depends on the tip voltage as

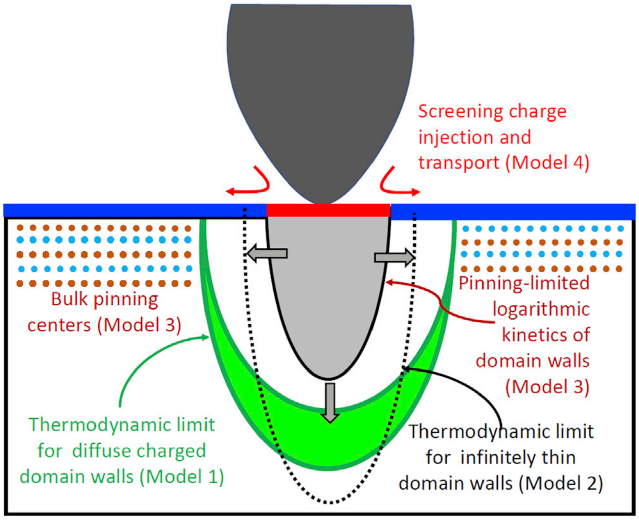

Figure 2. Schematics of the domain nucleation models

Model I shows thermodynamic limit for diffuse domain walls, which are thicker when becomes charged. Model II shows thermodynamic limit for infinitely thin domain walls. Model III is limited by domain wall pining in the bulk, giving rise to the logarithmic kinetics. Model IV is limited by the injection and transport of surface charged species that are necessary for the polarization screening.

$$r(V) \approx d\sqrt{(V/V_c)^{2/3} - 1}$$

, i.e., as the second-order phase transition scenario. Note that in the Bayesian setting, these models are similar and can be defined by tuning the prior distributions on parameter r_{cr}.

Model II corresponds to a Landauer-Molotskii (LM) approach of infinitely thin domain walls, very prolate domains, and their breakdown (see Figure 2).[60,61] For this case, the equilibrium domain radius depends on the tip voltage as

$$r(V) = r_{cr} + r_0\sqrt[3]{\left(\frac{V}{V_c}\right)^2 - 1}. \qquad \text{(Equation 2)}$$

Again, a jump V_c corresponds to the first-order phase transition and further growth as

$$\left(\frac{V}{V_c}\right)^{2/3}$$

.

Model III: alternative to the thermodynamic control of the domain size is the kinetic control, in which case the domain size is determined by the kinetics of the domain wall motion. From general theory of the disordered media, the domain wall velocity in the uniform field follows the classical dependence including pinning, creep, and then depinning and linear motion.[33,62–64] The available kinetic models[42,65] for the domain wall velocity $v(r)$ in a ferroelectric domain with pinning relate an acting electric field E, a threshold field E_{th}, and as $v(r) \approx v_0\, exp[-\ (E_{th}/E)^{\mu}]$, where μ is a positive exponential factor, which is typically close to unity. Using the simplest form for a normal component of the tip field, $E_z(r,0) = \frac{Vd^2}{\gamma(r^2+d^2)^{3/2}}$, where γ is a dielectric anisotropy factor, d is the effective tip size, r is a surface distance from the tip axis, and V is the bias applied between the tip and the bottom electrode, the approximate solution for the time dependence of the domain radius can be derived as (Appendix A)

$$r(t) \approx \left(\frac{V}{\beta}\right)^{1/3} ln\left[1 + \left(\frac{\beta}{V}\right)^{1/3} v_0 t\right], \qquad \text{(Equation 3)}$$

where the parameter $\beta = \gamma E_{th}/d^2$. Expression (4) describes a slow logarithmic creep of the domain wall such that $r(V) \sim V^{1/3}$ at high voltages. For $(\beta/V)^{1/3} v_0 t \gg 1$, we obtain $r(t) \sim (V/\beta)^{1/3} ln[v_0 t]$. However, the lateral growth stops at equilibrium domain sizes after the pulse ending, which can be calculated from thermodynamic description.

Finally, as model IV, we consider the case where the domain growth is limited by the transport of the screening charges across the sample surface. Here, we note that, in general, polarization switching requires almost complete compensation of the polarization charges by screening charges. If screening charges are abundant, the domain is determined by switching thermodynamics (model I and II) or wall pinning (model III). If the screening charges are slow and sparse, then the domain growth is limited by the charge injection. The experimental evidence toward this behavior was obtained by Yudin et al.[35] and also indirectly via observations of phenomena such as chaotic domain switching.[50,51]

The simple consideration of the mass and charge balance suggest that in the PFM experiment, the screening charges can be generated only at the tip-surface junction. In this case, assuming general power law voltage dependence of the generation rate and diffusional or drift transport of charge species, the kinetics of the domain wall growth can be described as

$$r(\tau) \approx V^{\alpha} \tau^{\beta}, \qquad \text{(Equation 4)}$$

where, in the depletion approximation, α is close to 1 and β is 1/2.

Experimental realization of hypothesis learning

As a model ferroelectric system, we use a fully relaxed 80 nm thick $BaTiO_3$ (BTO) thin film (see experimental procedures). A representative PFM image and domain writing are shown in Figure 3. Figure 3A is the surface topography showing uniform geometry with periodical terrace structures. Figures 3B and 3C are out-of-plane band-excitation PFM (BEPFM) amplitude and phase images of the original sample, respectively, and Figure 3D shows the corresponding resonance frequency related to local elastic property. The BTO film shows a down-polarized pristine state (Figure 3C). The polarization can be switched by applying a direct current (DC) bias via atomic force microscopy (AFM) tip. Shown in Figures 3E–3G are out-of-plane BEPFM amplitude, phase, and frequency images showing pre-poled areas by applying 5 V DC bias via AFM tip.

To realize hypothesis-learning-based automated experiment, we developed and deployed a workflow shown in Figure 4A integrating multiple software (including LabView, Jupyter Notebook, Google Colaboratory, and Igor) and hardware (including a National Instruments DAQ card, field-programmable gate arrays [FPGAs], and an Asylum Research Cypher microscope). To perform a BEPFM measurement, the measurement location (equivalent to

CellPress
OPEN ACCESS

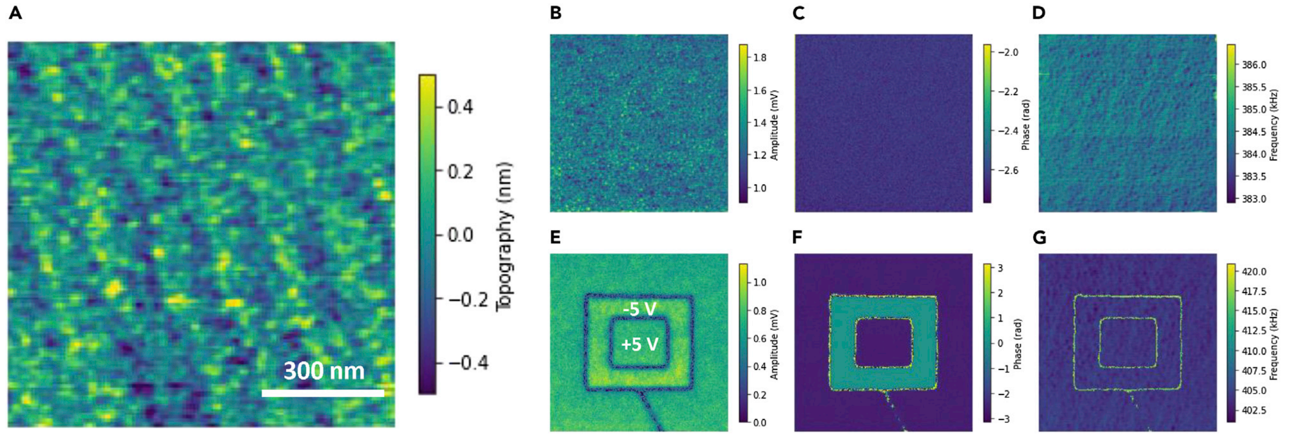

Figure 3. BEPFM of BTO sample

(A) Topography with periodical terrace structure.

(B–D) BEPFM amplitude, phase, and frequency images of the pristine state.

(E–G) BEPFM amplitude, phase, and frequency images of pre-poled areas by 5 V DC bias.

tip location) is controlled by FPGAs, and BEPFM data are acquired by the National Instruments DAQ card. To apply a pulse bias for writing domain, FPGA moves the tip to the target location (center of the experiment area) and applies the pulse bias to tip.

The workflow of hypothesis-learning automated BEPFM is shown in Figure 4B. The experiment starts with initializing the measurement area by applying a DC bias to uniformly pole this area toward the same direction. Then, domain writing and imaging are performed in this area. The acquired BEPFM data are analyzed by a threshold filter to detect the written domain, and the domain size is determined by the minimum closed circle in which the written domain lies either inside the circle or on its boundaries. Next, this domain size and writing parameters, as well as all previous domain sizes and writing parameters, are fed to the hypothesis-learning model to predict the next writing parameters. Simultaneously, when the hypothesis-learning algorithm is performing training and prediction, the workflow also controls the microscope to back switch the measurement area (erase the domain) in order to be ready for next writing iteration. Then, the predicted writing parameters will be fed to the measurement workflow, and the writing/imaging process will be performed again. For this workflow, we also added a function to check the BEPFM data quality such that a poor dataset will be discarded, and the same measurement will be repeated. This checking process ensures that all BEPFM data and, consequently, domain sizes are comparable.

For the hypotheses list, we have chosen four models following our discussion in mechanisms for ferroelectric domain growth. Equations corresponding to these models are shown in Table 1.

Shown in Figure 5 are the hypotheses-learning-based automated BEPFM results. In this experiment, 18 random writing parameters (5% of the writing parameters library) were selected to perform the initial domain writing experiment to provide initial ("seed") points for hypothesis learning. The obtained domain sizes along with corresponding writing parameters were used as initial training data for the hypothesis-learning algorithm. Then, 40 measurements were performed using writing parameters predicted by the algorithm. Figure 5A shows a few examples of the domains written in the BTO thin film, along with the binary domain images and domain size automatically detected by the workflow. It indicates that in this experiment, both the writing bias and writing time affect the domain size. In Figure 5B, all obtained results are shown as domain sizes as a function of write bias and time. Clearly, the larger bias and longer time result in increased domain sizes.

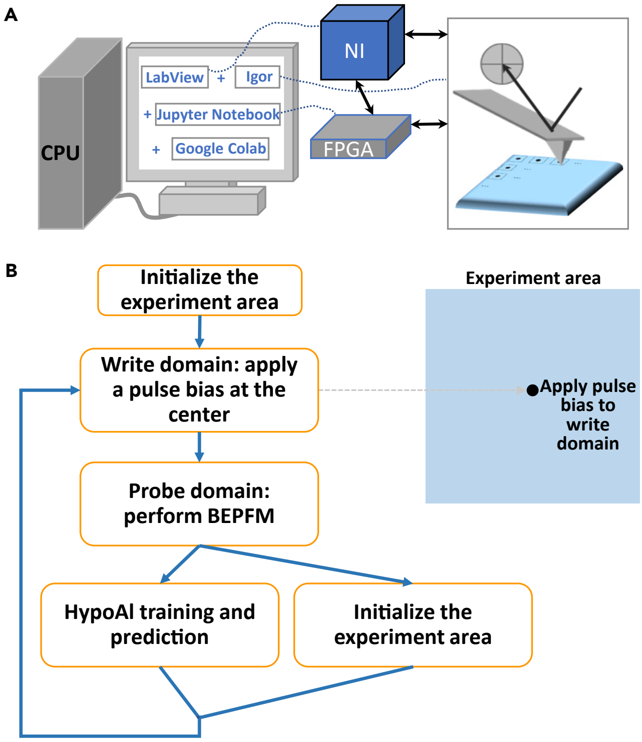

Figure 4. Hypothesis-learning automated BEPFM system and workflow

(A) A schematic showing the integrated system for hypothesis-learning automated BEPFM.

(B) Hypothesis-learning-based automated BEPFM workflow.

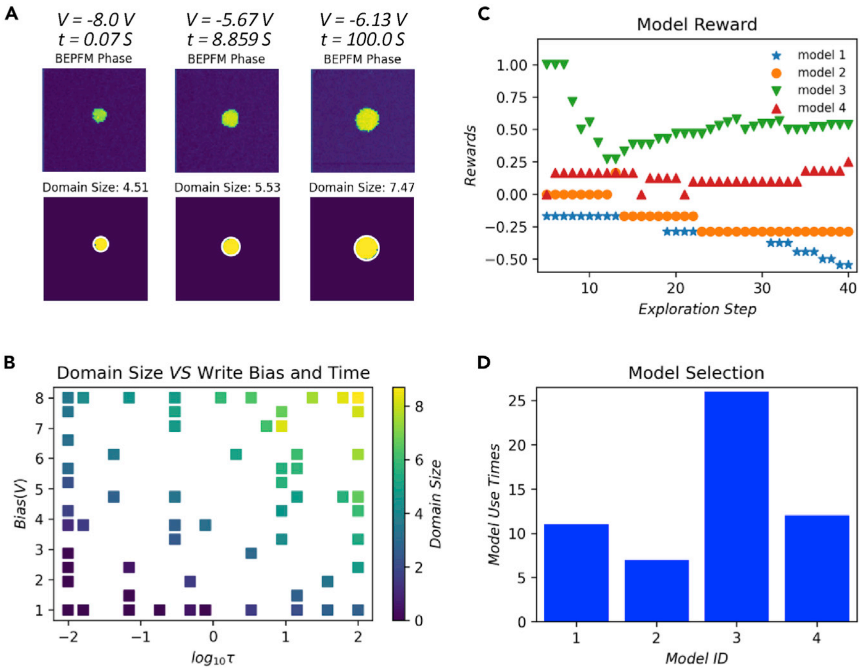

Figure 5. Hypotheses-learning-based automated BEPFM experiment results

(A) Three examples of domains written by using different bias and time. The top rows are BEPFM images showing the domains, and the bottom rows are corresponding binary images with domain size detected automatically by the automated workflow. Note that all BEPFM results and domain binary images are shown in Data S2 as a function of the measurement step.

(B) Domain size as a function of writing parameters.

(C) Model rewards during the hypotheses learning after the initial 5-step warmup phase, during which all models were evaluated at each step. Model III gained a much larger reward than other models, and its reward gradually increased at the latter part of the experiment.

(D) Model selection in hypotheses learning, in which model III was selected more often than other models.

Figure 5C shows the usage times of each model in the 40 step hypotheses learning, where the most often sampled model is model III. Figure 5D shows the evolution of rewards of each model. Model III "won" the most rewards, and its reward values steadily grow in the latter part of the experiment (this explains why it was selected more frequently than other models).

Shown in Figure 6 are predictions and uncertainties by four models after the experiment based on all obtained results. The predictions of models III and IV describe the experimental results (Figure 5B) better, and model III exhibits the lowest uncertainty, indicating that model III, meaning the domain wall pinning on the defect centers, is the correct one. These results suggest that the domain growth in this BTO thin film is determined by the kinetics of the domain wall motion rather than thermodynamics of the screening process, and the effect of surface screen charges is minor. In the automated experiment, the hypothesis learning also actively updates the model parameters. At the competition of the experiment, each model got parameters that best describe the experimental data. The final parameters of each model are summarized in Table 2.

Table 1. Model equations and priors used in the hypotheses-driven automated experiment

Model	Model equation	Model priors
I	$r(V) = r_{cr} + r_0\sqrt{\left(\frac{V}{V_c}\right)^{2/3} - 1}$	$r_{cr} \sim Normal(0,1)$<br>$r_0 \sim LogNormal(0,1)$<br>$V_c \sim LogNormal(0,1)$
II	$r(V) = r_{cr} + r_0\sqrt[3]{\left(\frac{V}{V_c}\right)^{2} - 1}$	$r_{cr} \sim Normal(0,1)$<br>$r_0 \sim LogNormal(0,1)$<br>$V_c \sim LogNormal(0,1)$
III	$r(V,t) = V^{\alpha} \log t$	$\alpha \sim Uniform(0.33, 1.2)$
IV	$r(V,t) = V^{\alpha} t^{\beta}$	$\alpha \sim Uniform(0.8, 1.2)$<br>$\beta \sim Uniform(0.33, 1.2)$

In all models, r is the domain radius ("size"), V is voltage applied through tip; t is the time of applied bias; and V_c, r_{cr}, r_0, α, and β are the model parameters, which will be inferred during experiments. More details about these models are available in Appendix S1.

Conclusions

To summarize, we have illustrated the hypothesis-learning-based automated experiment to explore domain switching. The hypothesis list has been built for different domain growth limiting stages, including thermodynamics of domain formation, domain wall pinning, and transport of screening charges at the surfaces. The results indicate that domain growth is ruled by kinetic control.

This approach for probing local bias-induced transformation is general and can be used for other tip-induced reactions and processes, including reversible and irreversible tip-induced electrochemical reactions including electroplating[66] and nanooxidation.[67,68] Note that the detection signal is not limited to the direct measurement of the domain size and can include measured currents, changes in topography, and resonance frequency shifts. As such, it can provide a powerful tool for probing neuromorphic materials[69] and fuel cell and battery materials,[70] as well as provide fundamental insights into electrochemical processes on the nanometer scale.[71]

We further note that the hypothesis learning can be broadly used in other automated experiment settings. Currently, this includes exploration of relatively low-dimensional parameter cases for which easy-to-evaluate competing physical models are available such as automated synthesis via microfluidic and robotic systems,[72] pulsed laser deposition, other forms of materials synthesis, etc.

EXPERIMENTAL PROCEDURES

Resource availability

Lead contact

Further information and requests for resources should be directed to and will be fulfilled by the lead contact, Yongtao Liu, liuy3@ornl.gov.

CellPress
OPEN ACCESS

Table 2. Final parameters of each model obtained from hypotheses-learning automated experiment

Model	Model equation	Final model parameters								
I	$r(V) = r_{cr} + r_0\sqrt{\left(\frac{V}{V_c}\right)^{2/3} - 1}$	r_{cr}			r_0			V_c		
		x̄	M	s	x̄	M	s	x̄	M	s
		−0.23	−0.25	0.89	2.35	2.37	0.72	0.53	0.53	0.24
II	$r(V) = r_{cr} + r_0\sqrt[3]{\left(\frac{V}{V_c}\right)^{2} - 1}$	r_{cr}			r_0			V_c		
		x̿	M	s	x̄	M	s	x̄	M	s
		0.06	0.84	0.02	1.03	1.03	0.36	0.65	0.59	0.40
III	$r(V,t) = V^{\alpha} \log t$	A								
		x̄			M			s		
		0.38			0.37			0.05		
IV	$r(V,t) = V^{\alpha} t^{\beta}$	A			β					
		x̄	M	s	x̄	M	s			
		0.81	0.81	0.01	0.33	0.33	0.00			

(1) x, mean value; M, median value; s, SD. (2) All model parameters are obtained from the last time a particular model was sampled in the hypotheses-learning experiment. Model I parameters are obtained from training step 38, measurement step 58; model II parameters are obtained from training step 22, measurement step 42; model III parameters are obtained from training step 40, measurement step 60; model IV parameters are obtained from training step 39, measurement step 59.

Materials availability

This study did not generate new materials.

Data and code availability

Code of this study is provided in the supplemental information.

Materials

BTO thin films were grown via pulsed laser deposition (PLD) in 99.9999% pure O_2 at 700°C. Specifically, first, a 5 nm $SrRuO_3$ back electrode was grown on (100) single-sided epitaxial-polished $SrTiO_3$ substrates at 100 millitorr with a pulse rate of 5 Hz from a stoichiometric $SrRuO_3$ ceramic target. Subsequently, 80 nm BTO was grown at 10 millitorr with a laser pulse rate of 10 Hz from a stoichiometric BTO target. The fluence for both thin-film layers was maintained at approximately 1.2 J/cm^2. Substrates were prepared by sonication in a warm (~70°C) deionized water bath for 1 min followed by an anneal at 1,000°C for 12 h to produce TiO_2 termination with step and terrace surface morphology.

Automated experiment in PFM

The hypothesis-learning-driven automated BEPFM measurement is based on an Asylum Research Cypher microscope equipped with a National Instruments DAQ card with LabView and a Field Programmable Gate Arrays with Python Jupyter Notebook. For domain writing, FPGA moves the tip to a desired location and applies a DC bias, followed by a BEPFM image measurement performed with NI, FPGA, and Cypher. The FPGA performs scan (move tip) and send to trigger to the NI DAQ card to perform BE measurements simultaneously. These processes are embedded in a Jupyter Notebook. When a measurement finishes, the Jupyter Notebook analyzes the BEPFM phase image to obtain the domain size and saves the domain size in Google Drive. Then, the hypothesis training is performed in Google Colaboratory with this domain size (and previous domain size), followed by saving the next writing parameters for the next experiment.

Hypothesis learning

The hypothesis learning (hypoAL)[27] was implemented using the home-build GPax package https://github.com/ziatdinovmax/gpax. The probabilistic models were wrapped into the structured Gaussian processes,[28] and the BI was performed via the iterative No-U-Turn sampler.[73] To ensure that wrapped models I and II remained isotropic in time, the kernel length scale for the time dimension was set to a sufficiently large value (1,000), whereas the kernel length scale for the voltage dimension was sampled from a standard weakly informative log-normal prior. For models III and IV, the ARD kernel in both dimensions was sampled from log-normal priors. The acquisition function value in each unmeasured point x_* was equal to the posterior predictive uncertainty

$$\mathbb{V}[f_*] = \frac{1}{N}\sum_{n=1}^{N}\left(f_*^n - \hat{f}_*\right)^2, \text{with } \hat{f}_* = \frac{1}{N}\sum_{n=1}^{N} P(x_*|\theta^n, D),$$

where $\theta^n \sim P(\theta|D)$ were samples drawn from the posterior and D was the available (measured) data. The reward function was defined as

$$R\left(\mathbb{V}_m^i, \mathbb{V}_m^{i-1}\right) = \begin{cases} +1, & \mathbb{V}_m^i < \mathbb{V}_m^{i-1} \\ -1, & \mathbb{V}_m^i \geq \mathbb{V}_m^{i-1} \end{cases},$$

where $\mathbb{V}_m^i$ is a median value of posterior predictive uncertainty at step i. The python script used to run the hypoAL during the experiment can be found in the supplemental information.

SUPPLEMENTAL INFORMATION

Supplemental information can be found online at https://doi.org/10.1016/j.patter.2023.100704.

ACKNOWLEDGMENTS

The authors acknowledge Stephen Jesse for helpful discussions and implementation of hardware-software integration and Vladimir A. Protopopescu for helpful discussions. This effort (physical hypotheses development, AE implementation, measurements) was supported as part of the center for 3D Ferroelectric Microelectronics (3DFeM), an Energy Frontier Research Center funded by the US Department of Energy (DOE), Office of Science, Basic Energy Sciences under award number DE-SC0021118. The research (hypothesis-learning development) was performed and partially supported at Oak Ridge National Laboratory's Center for Nanophase Materials Sciences (CNMS), a US DOE, Office of Science User Facility. A.N.M. was supported by the National Academy of Sciences of Ukraine and received funding from the European Union's Horizon 2020 research and innovation program under Marie Skłodowska-Curie grant agreement no. 778070. This manuscript has been authored by UT-Battelle, LLC, under contract no. DE-AC0500OR22725 with the US DOE. The United States government retains and the publisher, by accepting the article for publication, acknowledges that the United States Government retains a non-exclusive, paid-up, irrevocable, world-wide license to publish or reproduce the published form of this manuscript, or allow others to do so, for the United States government purposes. The DOE will provide public access to these results of federally sponsored research in accordance with the DOE Public Access Plan (http://energy.gov/downloads/doe-public-access-plan).

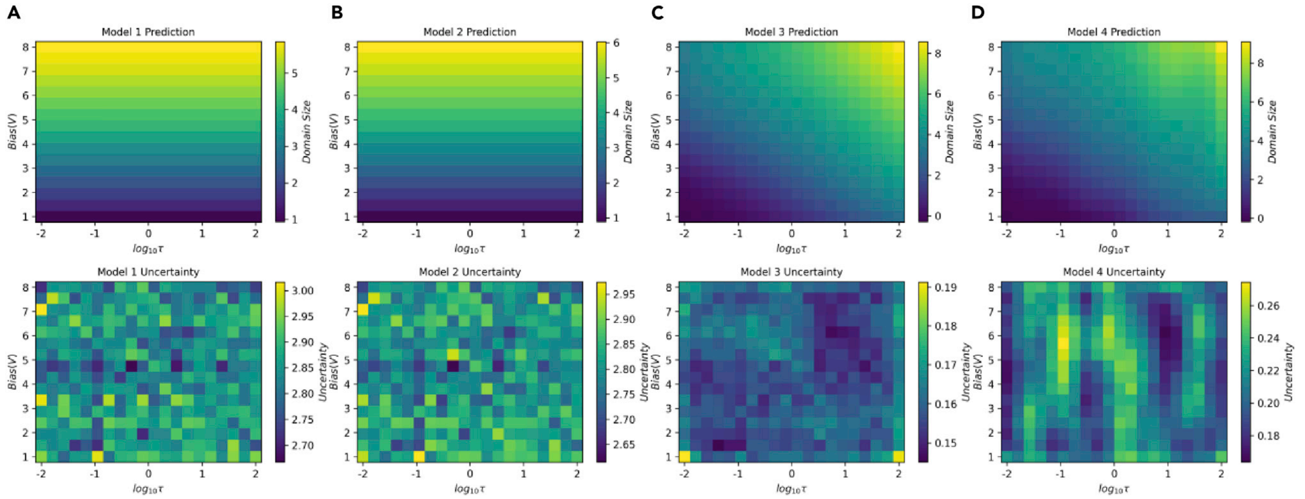

Figure 6. Predictions by all models on the final set of discovered parameters after the competition of the experiment
(A–D) Prediction (top row) and corresponding uncertainties (bottom row) by four different models, respectively.

AUTHOR CONTRIBUTIONS

Y.L. implemented the hypothesis-learning microscopy. Y.L. developed the hypothesis-learning microscope automation method with help from K.P.K. and R.V. Y.L. collected and analyzed the data. M.Z. realized the hypothesis-learning algorithms and provided the initial script. S.V.K. proposed and led the research. A.N.M. and E.A.E. developed the analytical models. All authors participated in the discussion.

DECLARATION OF INTERESTS

The authors have submitted a patent about hypothesis-learning-driven automated experiment.

INCLUSION AND DIVERSITY

We support inclusive, diverse, and equitable conduct of research.

Received: October 23, 2022
Revised: November 18, 2022
Accepted: February 9, 2023
Published: March 10, 2023

REFERENCES

1. Lemos, P., Jeffrey, N., Cranmer, M., Ho, S., and Battaglia, P. (2022). Rediscovering orbital mechanics with machine learning. Preprint at arXiv. https://doi.org/10.48550/arXiv.2202.02306.
2. Cranmer, M., Tamayo, D., Rein, H., Battaglia, P., Hadden, S., Armitage, P.J., Ho, S., and Spergel, D.N. (2021). A Bayesian neural network predicts the dissolution of compact planetary systems. Proc. Natl. Acad. Sci. USA *118*. e2026053118.
3. Albertsson, K., Altoe, P., Anderson, D., Andrews, M., Espinosa, J.P.A., Aurisano, A., Basara, L., Bevan, A., Bhimji, W., and Bonacorsi, D. (2018). Machine Learning in High Energy Physics Community White Paper (IOP Publishing), p. 022008.
4. De Luna, P., Wei, J., Bengio, Y., Aspuru-Guzik, A., and Sargent, E. (2017). Use Machine Learning to Find Energy Materials (Nature Publishing Group).
5. Mueller, T., Kusne, A.G., and Ramprasad, R. (2016). Machine learning in materials science: recent progress and emerging applications. Rev. Comput. Chem. *29*, 186–273.
6. Vasudevan, R.K., Choudhary, K., Mehta, A., Smith, R., Kusne, G., Tavazza, F., Vlcek, L., Ziatdinov, M., Kalinin, S.V., and Hattrick-Simpers, J. (2019). Materials science in the artificial intelligence age: high-throughput library generation, machine learning, and a pathway from correlations to the underpinning physics. MRS Commun. *9*, 821–838.
7. Ziatdinov, M., Dyck, O., Maksov, A., Li, X., Sang, X., Xiao, K., Unocic, R.R., Vasudevan, R., Jesse, S., and Kalinin, S.V. (2017). Deep learning of atomically resolved scanning transmission electron microscopy images: chemical identification and tracking local transformations. ACS Nano *11*, 12742–12752. https://doi.org/10.1021/acsnano.7b07504.
8. Zhu, J., Vuong, V.Q., Sumpter, B.G., and Irle, S. (2019). Artificial neural network correction for density-functional tight-binding molecular dynamics simulations. MRS Commun. *9*, 867–873.
9. Fung, V., Ganesh, P., and Sumpter, B.G. (2022). Physically informed machine learning prediction of electronic density of states. Chem. Mater. *34*, 4848–4855.
10. Kalinin, S.V., Ophus, C., Voyles, P.M., Erni, R., Kepaptsoglou, D., Grillo, V., Lupini, A.R., Oxley, M.P., Schwenker, E., Chan, M.K.Y., et al. (2022). Machine learning in scanning transmission electron microscopy. Nat. Rev. Methods Primers *2*, 11.
11. Wakabayashi, Y.K., Otsuka, T., Krockenberger, Y., Sawada, H., Taniyasu, Y., and Yamamoto, H. (2019). Machine-learning-assisted thin-film growth: Bayesian optimization in molecular beam epitaxy of SrRuO3 thin films. Apl. Mater. *7*, 101114.
12. Ovchinnikov, O.S., Jesse, S., Bintacchit, P., Trolier-McKinstry, S., and Kalinin, S.V. (2009). Disorder identification in hysteresis data: recognition analysis of the random-bond-random-field ising model. Phys. Rev. Lett. *103*, 157203. https://doi.org/10.1103/PhysRevLett.103.157203.
13. Fischer, C.C., Tibbetts, K.J., Morgan, D., and Ceder, G. (2006). Predicting crystal structure by merging data mining with quantum mechanics. Nat. Mater. *5*, 641–646. https://doi.org/10.1038/nmat1691.
14. Krizhevsky, A., Sutskever, I., and Hinton, G.E. (2012). ImageNet classification with deep convolutional neural networks. Adv. Neural Inf. Process. Syst. *25* NIPS 2012.
15. DALLE.
16. ChatGPT.
17. Krull, A., Hirsch, P., Rother, C., Schiffrin, A., and Krull, C. (2020). Artificial-intelligence-driven scanning probe microscopy. Commun. Phys. *3*, 54. https://doi.org/10.1038/s42005-020-0317-3.
18. Noack, M.M., Doerk, G.S., Li, R., Fukuto, M., and Yager, K.G. (2020). Advances in kriging-based autonomous X-ray scattering experiments. Sci. Rep. *10*, 1325. https://doi.org/10.1038/s41598-020-57887-x.
19. Noack, M.M., Yager, K.G., Fukuto, M., Doerk, G.S., Li, R., and Sethian, J.A. (2019). A kriging-based approach to autonomous experimentation

with applications to X-ray scattering. Sci. Rep. *9*, 11809. https://doi.org/10.1038/s41598-019-48114-3.

20. Nikolaev, P., Hooper, D., Perea-López, N., Terrones, M., and Maruyama, B. (2014). Discovery of wall-selective carbon nanotube growth conditions via automated experimentation. ACS Nano *8*, 10214–10222. https://doi.org/10.1021/nn503347a.
21. MacLeod, B.P., Parlane, F.G.L., Morrissey, T.D., Häse, F., Roch, L.M., Dettelbach, K.E., Moreira, R., Yunker, L.P.E., Rooney, M.B., Deeth, J.R., et al. (2020). Self-driving laboratory for accelerated discovery of thin-film materials. Sci. Adv. *6*, eaaz8867. https://doi.org/10.1126/sciadv.aaz8867.
22. Epps, R.W., Bowen, M.S., Volk, A.A., Abdel-Latif, K., Han, S., Reyes, K.G., Amassian, A., and Abolhasani, M. (2020). Artificial chemist: an autonomous quantum dot synthesis bot. Adv. Mater. *32*, e2001626. https://doi.org/10.1002/adma.202001626.
23. Higgins, K., Valleti, S.M., Ziatdinov, M., Kalinin, S.V., and Ahmadi, M. (2020). Chemical robotics enabled exploration of stability in multicomponent lead halide perovskites via machine learning. ACS Energy Lett. *5*, 3426–3436. https://doi.org/10.1021/acsenergylett.0c01749.
24. Martin, O. (2018). Bayesian Analysis with Python: Introduction to Statistical Modeling and Probabilistic Programming Using PyMC3 and ArviZ, 2nd Edition (Packt Publishing).
25. Lambert, B. (2018). A Student's Guide to Bayesian Statistics, 1 edition (SAGE Publications Ltd).
26. Kruschke, J. (2014). Doing Bayesian Data Analysis: A Tutorial with R, JAGS, and Stan, 2 edition (Academic Press).
27. Ziatdinov, M., Liu, Y., Morozovska, A.N., Eliseev, E.A., Zhang, X., Takeuchi, I., and Kalinin, S.V. (2021). Hypothesis learning in an automated experiment: application to combinatorial materials libraries. Preprint at arXiv. https://doi.org/10.48550/arXiv.2112.06649.
28. Ziatdinov, M., Ghosh, A., and Kalinin, S.V. (2022). Physics makes the difference: Bayesian optimization and active learning via augmented Gaussian process. Mach. Learn.: Sci. Technol.
29. Kolosov, O., Gruverman, A., Hatano, J., Takahashi, K., and Tokumoto, H. (1995). Nanoscale visualization and control of ferroelectric domains by atomic-force microscopy. Phys. Rev. Lett. *74*, 4309–4312. https://doi.org/10.1103/PhysRevLett.74.4309.
30. Gruverman, A., Auciello, O., Ramesh, R., and Tokumoto, H. (1997). Scanning force microscopy of domain structure in ferroelectric thin films: imaging and control. Nanotechnology *8*, A38–A43. https://doi.org/10.1088/0957-4484/8/3a/008.
31. Alexe, M., Gruverman, A., Harnagea, C., Zakharov, N.D., Pignolet, A., Hesse, D., and Scott, J.F. (1999). Switching properties of self-assembled ferroelectric memory cells. Appl. Phys. Lett. *75*, 1158–1160. https://doi.org/10.1063/1.124628.
32. Rodriguez, B.J., Nemanich, R.J., Kingon, A., Gruverman, A., Kalinin, S.V., Terabe, K., Liu, X.Y., and Kitamura, K. (2005). Domain growth kinetics in lithium niobate single crystals studied by piezoresponse force microscopy. Appl. Phys. Lett. *86*, 012906. https://doi.org/10.1063/1.1844594.
33. Tybell, T., Paruch, P., Giamarchi, T., and Triscone, J.M. (2002). Domain wall creep in epitaxial ferroelectric Pb(Zr(0.2)Ti(0.8))O(3) thin films. Phys. Rev. Lett. *89*, 097601. https://doi.org/10.1103/PhysRevLett.89.097601.
34. Woo, J., Hong, S., Setter, N., Shin, H., Jeon, J.U., Pak, Y.E., and No, K. (2001). Quantitative analysis of the bit size dependence on the pulse width and pulse voltage in ferroelectric memory devices using atomic force microscopy. J. Vac. Sci. Technol. B. *19*, 818–824. https://doi.org/10.1116/1.1364697.
35. Yudin, P.V., Hrebtov, M.Y., Dejneka, A., and McGilly, L.J. (2020). Modeling the motion of ferroelectric domain walls with the classical Stefan problem. Phys. Rev. Appl. *13*, 014006.
36. Molotskii *, M.I., and Shvebelman, M.M. (2005). Dynamics of ferroelectric domain formation in an atomic force microscope. Phil. Mag. *85*, 1637–1655. https://doi.org/10.1080/14786430312331524670.
37. Kalinin, S.V., Gruverman, A., Rodriguez, B.J., Shin, J., Baddorf, A.P., Karapetian, E., and Kachanov, M. (2005). Nanoelectromechanics of polarization switching in piezoresponse force microscopy. J. Appl. Phys. *97*, 074305. https://doi.org/10.1063/1.1866483.
38. Morozovska, A.N., Kalinin, S.V., Eliseev, E.A., and Svechnikov, S.V. (2007). Local polarization switching in piezoresponse force microscopy. Ferroelectrics *354*, 198–207. https://doi.org/10.1080/00150190701454966.
39. Morozovska, A.N., Eliseev, E.A., Li, Y., Svechnikov, S.V., Maksymovych, P., Shur, V.Y., Gopalan, V., Chen, L.Q., and Kalinin, S.V. (2009). Thermodynamics of nanodomain formation and breakdown in scanning probe microscopy: Landau-Ginzburg-Devonshire approach. Phys. Rev. B *80*, 214110. https://doi.org/10.1103/PhysRevB.80.214110.
40. Aravind, V.R., Morozovska, A.N., Bhattacharyya, S., Lee, D., Jesse, S., Grinberg, I., Li, Y.L., Choudhury, S., Wu, P., Seal, K., et al. (2010). Correlated polarization switching in the proximity of a 180 degrees domain wall. Phys. Rev. B *82*, 024111. https://doi.org/10.1103/PhysRevB.82.024111.
41. Kalinin, S.V., Morozovska, A.N., Chen, L.Q., and Rodriguez, B.J. (2010). Local polarization dynamics in ferroelectric materials. Rep. Prog. Phys. *73*, 056502. https://doi.org/10.1088/0034-4885/73/5/056502.
42. Molotskii, M. (2005). Generation of ferroelectric domains in films using atomic force microscope. J. Appl. Phys. *97*, 014109.
43. Son, J.Y., Shin, Y.H., Lee, H.B.R., Kim, H., Cho, J.H., and Ali, A.I. (2007). Surface charge on ferroelectric thin film by high electric field induced at scanning probe microscope tip. J. Kor. Phys. Soc. *51*, S125–S128.
44. Cunningham, S., Larkin, I.A., and Davis, J.H. (1998). Noncontact scanning probe microscope potentiometry of surface charge patches: origin and interpretation of time-dependent signals. Appl. Phys. Lett. *73*, 123–125. https://doi.org/10.1063/1.121788.
45. Bühlmann, S., Colla, E., and Muralt, P. (2005). Polarization reversal due to charge injection in ferroelectric films. Phys. Rev. B *72*, 214120. https://doi.org/10.1103/PhysRevB.72.214120.
46. Kim, Y., Bühlmann, S., Hong, S., Kim, S.H., and No, K. (2007). Injection charge assisted polarization reversal in ferroelectric thin films. Appl. Phys. Lett. *90*, 072910. https://doi.org/10.1063/1.2679902.
47. Kim, Y., Kim, J., Bühlmann, S., Hong, S., Kim, Y.K., Kim, S.H., and No, K. (2008). Screen charge transfer by grounded tip on ferroelectric surfaces. Phys. Status Solidi RRL *2*, 74–76. https://doi.org/10.1002/pssr.200701265.
48. Brugère, A., Gidon, S., and Gautier, B. (2011). Abnormal switching of ferroelectric domains created by the tip of an atomic force microscope in a congruent LiTaO(3) single-crystal thin film. J. Appl. Phys. *110*, 024102. https://doi.org/10.1063/1.3607302.
49. Li, Q., Liu, Y., Schiemer, J., Smith, P., Li, Z., Withers, R.L., and Xu, Z. (2011). Fully-inverted piezoresponse hysteresis loops mediated by charge injection in 0.29Pb(In(1/2)Nb(1/2))O(3)-0.44Pb(Mg(1/3)Nb(2/3))O(3)-0.27PbTiO(3) single crystals. Appl. Phys. Lett. *98*, 092908. https://doi.org/10.1063/1.3562034.
50. Ievlev, A.V., Jesse, S., Morozovska, A.N., Strelcov, E., Eliseev, E.A., Pershin, Y.V., Kumar, A., Shur, V.Y., and Kalinin, S.V. (2014). Intermittency, quasiperiodicity and chaos in probe-induced ferroelectric domain switching. Nat. Phys. *10*, 59–66. https://doi.org/10.1038/nphys2796.
51. Ievlev, A.V., Morozovska, A.N., Eliseev, E.A., Shur, V.Y., and Kalinin, S.V. (2014). Ionic field effect and memristive phenomena in single-point ferroelectric domain switching. Nat. Commun. *5*, 4545. https://doi.org/10.1038/ncomms5545.
52. Hlinka, J., and Ondrejkovic, P. (2019). Skyrmions in ferroelectric materials. Solid State Phys. *70*, 143–169.
53. Gruverman, A., Alexe, M., and Meier, D. (2019). Piezoresponse force microscopy and nanoferroic phenomena. Nat. Commun. *10*, 1661.
54. Wang, J.-J., Wang, B., and Chen, L.-Q. (2019). Understanding, predicting, and designing ferroelectric domain structures and switching guided by the phase-field method. Annu. Rev. Mater. Res. *49*, 127–152.
55. Tagantsev, A.K., Cross, L.E., and Fousek, J. (2010). Domains in Ferroic Crystals and Thin Films (Springer).

56. Woo, J., Hong, S., Min, D.K., Shin, H., and No, K. (2002). Effect of domain structure on thermal stability of nanoscale ferroelectric domains. Appl. Phys. Lett. *80*, 4000–4002.
57. Morozovska, A.N., Eliseev, E.A., Li, Y., Svechnikov, S.V., Maksymovych, P., Shur, V.Y., Gopalan, V., Chen, L.-Q., and Kalinin, S.V. (2009). Thermodynamics of nanodomain formation and breakdown in scanning probe microscopy: Landau-Ginzburg-Devonshire approach. Phys. Rev. B *80*, 214110.
58. Chen, J., Gruverman, A., Morozovska, A.N., and Valanoor, N. (2014). Sub-critical field domain reversal in epitaxial ferroelectric films. J. Appl. Phys. *116*, 124109.
59. Panwar, N., Bdikin, I.K., Morozovska, A.N., and Kholkin, A.L. (2012). Domain growth kinetics in La0. 89Sr0. 11MnO3 single crystal studied by piezoresponse force microscopy. J. Appl. Phys. *112*, 052019.
60. Molotskii, M., Agronin, A., Urenski, P., Shvebelman, M., Rosenman, G., and Rosenwaks, Y. (2003). Ferroelectric domain breakdown. Phys. Rev. Lett. *90*, 107601.
61. Molotskii, M. (2003). Generation of ferroelectric domains in atomic force microscope. J. Appl. Phys. *93*, 6234–6237.
62. Pertsev, N.A., Kiselev, D.A., Bdikin, I.K., Kosec, M., and Kholkin, A.L. (2011). Quasi-one-dimensional domain walls in ferroelectric ceramics: evidence from domain dynamics and wall roughness measurements. J. Appl. Phys. *110*, 052001. https://doi.org/10.1063/1.3624810.
63. Paruch, P., Giamarchi, T., and Triscone, J.M. (2007). Nanoscale studies of domain walls in epitaxial ferroelectric thin films. In Physics of Ferroelectrics: A Modern Perspective, K.M. Rabe, C.H. Ahn, and J.M. Triscone, eds. (Springer-Verlag Berlin), pp. 339–362.
64. Nattermann, T., Pokrovsky, V., and Vinokur, V.M. (2001). Hysteretic dynamics of domain walls at finite temperatures. Phys. Rev. Lett. *87*, 197005.
65. Paruch, P., Giamarchi, T., Tybell, T., and Triscone, J.-M. (2006). Nanoscale studies of domain wall motion in epitaxial ferroelectric thin films. J. Appl. Phys. *100*, 051608.
66. Arruda, T.M., Kumar, A., Kalinin, S.V., and Jesse, S. (2011). Mapping irreversible electrochemical processes on the nanoscale: ionic phenomena in Li ion conductive glass ceramics. Nano Lett. *11*, 4161–4167. https://doi.org/10.1021/nl202039v.
67. García, R., Calleja, M., and Pérez-Murano, F. (1998). Local oxidation of silicon surfaces by dynamic force microscopy: nanofabrication and water bridge formation. Appl. Phys. Lett. *72*, 2295–2297.
68. Tello, M., and García, R. (2001). Nano-oxidation of silicon surfaces: comparison of noncontact and contact atomic-force microscopy methods. Appl. Phys. Lett. *79*, 424–426. https://doi.org/10.1063/1.1385582.
69. Szot, K., Rogala, M., Speier, W., Klusek, Z., Besmehn, A., and Waser, R. (2011). TiO(2)-a prototypical memristive material. Nanotechnology *22*, 254001. https://doi.org/10.1088/0957-4484/22/25/254001.
70. Kostecki, R., and McLarnon, F. (2000). Nanometer-scale electrochemical lithography on the spinel oxide LiMn2O4. Appl. Phys. Lett. *76*, 2535–2537. https://doi.org/10.1063/1.126400.
71. Garcia, R., Losilla, N.S., Martínez, J., Martinez, R.V., Palomares, F.J., Huttel, Y., Calvaresi, M., and Zerbetto, F. (2010). Nanopatterning of carbonaceous structures by field-induced carbon dioxide splitting with a force microscope. Appl. Phys. Lett. *96*, 143110. https://doi.org/10.1063/1.3374885.
72. Langner, S., Häse, F., Perea, J.D., Stubhan, T., Hauch, J., Roch, L.M., Heumueller, T., Aspuru-Guzik, A., and Brabec, C.J. (2020). Beyond ternary OPV: high-throughput experimentation and self-driving laboratories optimize multicomponent systems. Adv. Mater. *32*, 1907801. https://doi.org/10.1002/adma.201907801.
73. Phan, D., Pradhan, N., and Jankowiak, M. (2019). Composable effects for flexible and accelerated probabilistic programming in NumPyro. Preprint at arxiv. https://doi.org/10.48550/arXiv.1912.11554.

Patterns, Volume *4*

Supplemental information

Autonomous scanning probe microscopy with hypothesis learning: Exploring the physics of domain switching in ferroelectric materials

Yongtao Liu, Anna N. Morozovska, Eugene A. Eliseev, Kyle P. Kelley, Rama Vasudevan, Maxim Ziatdinov, and Sergei V. Kalinin

Supplemental Experimental Procedures

Autonomous Scanning Probe Microscopy with Hypothesis Learning: Exploring the Physics of Domain Switching in Ferroelectric Materials

Yongtao Liu,[1, a, *] Anna Morozovska,[2] Eugene Eliseev,[2,3] Kyle P. Kelley,[1] Rama Vasudevan,[1] Maxim Ziatdinov,[1,4,b] and Sergei V. Kalinin[1, c]

1 Center for Nanophase Materials Sciences, Oak Ridge National Laboratory, Oak Ridge, TN 37922, United States

2 Institute of Physics, National Academy of Sciences of Ukraine, 46, pr. Nauky, 03028 Kyiv, Ukraine

3 Institute for Problems of Materials Science, National Academy of Sciences of Ukraine, Krjijanovskogo 3, 03142 Kyiv, Ukraine

4 Computational Sciences and Engineering Division, Oak Ridge National Laboratory, Oak Ridge, TN 37831, United States

[a, b, c] Corresponding authors' email addresses: liuy3@ornl.gov ; ziatdinovma@ornl.gov ; sergei2@utk.edu

*Lead Contact's email address: liuy3@ornl.gov

1. **Supplementary Appendix A.** Models of domain growth
2. **Supplementary Note.** Challenges to Implementing Machine Learning-Driven Autonomous Microscopy
3. **Data S1.** Python script used to run the hypoAL during the experiment
4. **Data S2.** PFM phase image, binary image showing domain size, domain size as a function of write parameters, model selection, and model reward as a function of measurement step.

1. Appendix A. Models of domain growth

A. Thermodynamic models 1-3

The voltage dependences of the equilibrium domain sizes are listed in **Table I**. They were selected based on Refs. [i, ii, iii].

Table I. Adapted from Ref. [i]

**Domain charac-teristics**	**Intrinsic model of domain formation for thick films** ($h >> \gamma d$)		
	**Case (i)**: the complete screening of depolarization field	**Case (ii)**: no screening of depolarization field, but the wall can be thick	**Case (iii):** LM- model infinitely thin domain walls
Coercive bias V_c	$V_c = \gamma d \cdot E_c$	$V_c \approx \gamma d(E_c + \Delta E)$, $\Delta E = 2P_S/(\varepsilon_0\varepsilon_{11}\gamma^2)$	Analytical expression for V_c is thickness-dependent
Domain onset at $V \to V_c$	Onset is the second order phase transition, i.e., activationless. An oblate domain appears with very small sizes $r(V_c) = 0$ and $l(V_c) \sim r^2(V_c)$.	Onset is the first order phase transition, i.e., the barrier should exist. Nucleus is very prolate and spike-like $r(V_c) \ll l(V_c)$, and rapidly growth downward.	Activation barrier exists for the length, since prolate stable domain appears with nonzero sizes $l(V_c) > r(V_c) > 0$
Sizes vs. bias V, at $V \geq V_c$	$r(V) = d\sqrt{\left(\frac{V}{V_c}\right)^{2/3} - 1}$ $l(V) = \gamma \cdot d\sqrt{\frac{V}{V_c} - 1}$	$r(V) \approx r_{cr} + d\sqrt{\left(\frac{V}{V_c}\right)^{2/3} - 1}$ $l(V) = l_{cr} + l_0\sqrt{\frac{V}{V_c} - 1}$ length $l >> r$ is determined by depolarization energy value.	$r(V) = r_{cr} + r_0\sqrt[3]{\left(\frac{V}{V_c}\right)^{2} - 1}$ $l(V) = l_{cr} + l_0\sqrt{\left(\frac{V}{V_c}\right)^{2} - 1}$ length $l >> r$ is determined by the interplay between the depolarization energy and domain wall surface energy.
Sizes vs. bias V, at $V \gg V_c$	$r(V) \sim V^{1/3}$ $l(V) \sim V^{1/2}$ $r^3/l^2 \approx \gamma^{-2}$	$r(V) \sim V^{1/3}$ $l(V) \sim V^{1/2}$ $r^3/l^2 \approx \gamma^{-2}$	$r(V) \sim V^{2/3}$ $l(V) \sim V$ $r^3/l^2 \approx \text{const}$

γ is a dielectric anisotropy factor

B. Kinetic model 4

The domain wall velocity in a ferroelectric containing defects is related to an acting electric field E as [iv, v]

$$v(r) \approx v_0\, exp\left[-\left(\frac{E_{th}}{E}\right)^{\mu}\right] \qquad (1a)$$

Where μ is a positive number, so-called exponential factor, that is typically close to unity. E_{th} is the activation field, defined as the threshold field, above which the domain wall is unpinned

by defects. Another situation corresponds to a rapid wall motion (in very high fields) without pinning effects:

$$v(r) \approx v_0 \left(\frac{E}{E_{cr}}\right)^{\chi}, \qquad E \gg E_{cr} \tag{1b}$$

Using the simplest form for a normal component of the tip field,

$$E_z(r,0) = \frac{Vd^2}{\gamma(r^2+d^2)^{3/2}}, \tag{2}$$

and ignore the depolarization field in Eq.(1a), we obtain:

$$v(r) \approx v_0\, exp\left[-\left(\frac{\gamma E_{th}}{Vd^2}\sqrt{(r^2+d^2)^3}\right)^{\mu}\right] \tag{3a}$$

Here γ is a dielectric anisotropy factor, r is a surface distance from the tip axis, and V is the bias applied between the tip and the bottom electrode. So, the velocity at the domain boundary depends on the domain radius. Note that one can neglect the depolarization field under specific conditions, such as the lateral growth of uncharged domain wall, or arbitrary growth accompanied by a local screening of depolarization field by free carriers. However, the screening carriers should also affect on the tip field, so the second case is exotic.

For the case $E \gg E_{cr}$

$$v(r) \approx v_0 \left(\frac{Vd^2}{\gamma E_{cr}(r^2+d^2)^{3/2}}\right)^{\chi}, \tag{3b}$$

Where the factor χ is positive.

The instant domain radius is $r(t) = \int_0^t v(t')dt'$, since $\frac{dr}{dt} = v$. So, to define the domain wall velocity we need to solve a differential equation:

$$\frac{dr}{dt} \approx v_0\, exp\left[-\left(\frac{\gamma E_{th}}{Vd^2}\sqrt{(r^2+d^2)^3}\right)^{\mu}\right], \tag{4a}$$

or, for the case $E \gg E_{cr}$,

$$\frac{dr}{dt} \approx v_0 \left(\frac{Vd^2}{\gamma E_{cr}(r^2+d^2)^{3/2}}\right)^{\chi}, \tag{4b}$$

Eq.(4a) has no simple analytical solution. Putting $\mu = 1/3$ and regarding that we are interested in the case $r \gg d$, we obtain that $v_0 t = \left(\frac{Vd^2}{\gamma E_{th}}\right)^{1/3} \left(exp\left[\left(\frac{\gamma E_{th}}{Vd^2}\right)^{1/3} r\right] - 1\right)$. So, the approximate solution is

$$r(t) \approx \left(\frac{Vd^2}{\gamma E_{th}}\right)^{1/3} ln\left[1 + \left(\frac{\gamma E_{th}}{Vd^2}\right)^{1/3} v_0 t\right]. \tag{5a}$$

Expression (5a) describes a slow logarithmic creep of the domain wall, at that $r(V)\sim V^{1/3}$ at high voltages. Actually at $\left(\frac{\gamma E_{th}}{Vd^2}\right)^{1/3} v_0 t \gg 1$, we obtain that $r(t)\sim \left(\frac{Vd^2}{\gamma E_{th}}\right)^{1/3} ln[v_0 t]$. However,

the lateral growth stops at equilibrium domain sizes after the pulse ending, which can be calculated from thermodynamic description.

Eq.(4b) has a simple analytical solution. Being interested in the case $r \gg d$, we can obtain different power laws:

$$r(t) \approx \frac{1}{3\chi+1}\left[v_0 t\left(\frac{V}{\gamma E_{cr} d}\right)^{\chi}\right]^{\frac{1}{3\chi+1}} \sim V^{\frac{\chi}{3\chi+1}}(v_0 t)^{\frac{1}{3\chi+1}}. \qquad (5b)$$

Note that the opposite case $r \ll d$ seems to be unphysical, since it means very small times. However, Eq.(5b) states that at positive χ, the domain growth is sub-linear both in voltage and it time.

The intrinsic coercive field E_c is well-known, and can be expressed via the LGD functional expansion coefficients as [vi]:

$$E_c = \begin{cases} \frac{2}{3\sqrt{3}}\sqrt{-\frac{\alpha^3}{\beta}} \text{ ,for second order ferroelectrics,} \\ \frac{2}{5}\left(2\beta + \sqrt{9\beta^2 - 20\alpha\delta}\right)\left(\frac{2\alpha}{-3\beta-\sqrt{9\beta^2-20\alpha\delta}}\right)^{3/2} \text{ for first order ferroelectrics.} \end{cases} \qquad (6)$$

Note that expressions listed in Table I for the case of the electric excitation by the localized probe field with characteristic scale d differs from the semi-empirical Kay-Dunn law [vii], which stated that $r \sim h^{2/3}$and coercive field $E_{cr} \sim h^{-2/3}$ for homogeneous external field.

2. Supplementary Note. Challenges to Implementing Machine Learning-Driven Autonomous Microscopy

Nowadays, most commercialized SPM microscopes do not allow programmatic control (e.g. control by Python program), so the first challenge is to enable the automation of microscopes. This requires lots of effort to develop electronics and software in order to control the microscope through Python program. In our work, we used an FPGA and developed a script that allows us to send voltage to (1) the piezo scanner of the microscope, and (2) the tip; so that we can drive the tip to perform PFM scan and apply a voltage to the tip to write a domain. Second, multiple software and Python programs are used for this experiment, these software and programs need different operating systems. For instance, in this experiment, hypothesis learning requires a Linux environment but other software requires a Windows environment. Thus, in order to perform real-time hypothesis learning during measurement, we need to transfer experiment results and hypothesis learning analysis results between Linux and Windows computers in real-time. Here, we used an ethernet cable to enable real-time data transfer. However, we would like to note that these challenges and corresponding solutions may vary in different labs, depending on microscopes and lab conditions.

References

[i] A.N. Morozovska, E.A. Eliseev, Yulan Li, S.V. Svechnikov, P. Maksymovych, V.Y. Shur, Venkatraman Gopalan, Long-Qing Chen, and S.V. Kalinin. Thermodynamics of nanodomain formation and breakdown in Scanning Probe Microscopy: Landau-Ginzburg-Devonshire approach. Phys. Rev. B. 80, 214110 (2009).

[ii] Jason Chen, Alexei Gruverman, Anna N. Morozovska and Nagarajan Valanoor. Sub-Critical Field Domain Reversal in Epitaxial Ferroelectric Films. J. Appl. Phys. **116**, 124109 (2014); http://dx.doi.org/10.1063/1.4896730

[iii] N. Panwar, I. K. Bdikin, A.N. Morozovska, and A. L. Kholkin, J. Appl. Phys **112**, 052019 (2012)

[iv] M. Molotskii, J. Appl. Phys. **97**, 6234 (2005).

[v] P. Paruch, T. Giamarchi, T. Tybell, and J. M. Triscone, J. Appl. Phys. **100**, 051608 (2006).

[vi] S. Ducharme, V. M. Fridkin, A.V. Bune, S. P. Palto, L. M. Blinov, N. N. Petukhova, S. G. Yudin, Phys. Rev. Lett. **84**, 175 (2000).

[vii] H.F. Kay and J.W. Dunn, Phil. Mag. **7**, 2027 (1962).
